# Porphyrin-based zinc metal–organic framework loaded with gallic acid as a novel nanoplatform exhibiting H_2_O_2_-activated reactive oxygen species generation and cytotoxicity in breast cancer cells

**DOI:** 10.1039/d5ra09585a

**Published:** 2026-03-02

**Authors:** May R. Ibrahim, Shaikha Alneyadi, Hesham El-Maghraby, Stefan Wuttke, Yaser Greish

**Affiliations:** a Department of Chemistry, UAE University Al Ain 15551 United Arab Emirates y.afifi@uaeu.ac.ae; b Academic Centre for Materials and Nanotechnology, AGH University of Krakow Krakow 30-059 Poland; c Zayed Centre for Health Sciences, United Arab Emirates University Al-Ain 15551 United Arab Emirates

## Abstract

A zinc-based metal–organic framework (MOF) engineered from tetrakis(4-carboxyphenyl) porphyrin (TCPP) as an organic linker and functionalized with gallic acid (GA) as an active therapeutic agent demonstrates remarkable potential for cancer treatment. The resulting Zn-TCPP@GA hybrid framework exhibits a high specific surface area, extensive π–π conjugation, and superior catalytic performance, collectively facilitating efficient reactive oxygen species (ROS) generation-an essential mechanism underlying chemodynamic therapy (CDT). Incorporation of GA significantly enhances the redox activity and biocompatibility of the framework. GA actively participates in modulating the tumor environment by depleting intracellular glutathione (GSH), thereby impairing the antioxidant defense machinery of cancer cells and amplifying ROS-mediated oxidative stress. Comprehensive physicochemical characterization confirmed that Zn-TCPP@GA exhibits an intrinsic peroxidase-mimetic and ROS generation mechanism *via* catalyzing the decomposition of hydrogen peroxide (H_2_O_2_) into highly reactive hydroxyl radicals (˙OH). This catalytic conversion markedly augments intracellular ROS accumulation, resulting in pronounced oxidative damage and selective cytotoxicity toward malignant cells while sparing normal tissues. *In vitro* cytotoxicity evaluation revealed that Zn-TCPP@GA at a concentration of 75.04 µg mL^−1^ induced approximately a 50% reduction in MCF-7 breast cancer cell viability, with negligible impact on normal cell lines. Collectively, these findings substantiate Zn-TCPP@GA as a potent CDT nanotherapeutic platform, capable of tumor-selective ROS amplification through peroxidase-like catalysis and chemodynamic biochemical modulation mediated by gallic acid.

## Abstract 2

A novel zinc-based 2-dimensional porphyrin metal–organic framework (MOF), and further functionalized with gallic acid (GA) as an active therapeutic agent demonstrates remarkable potential for anticancer applications.

## Graphical abstract

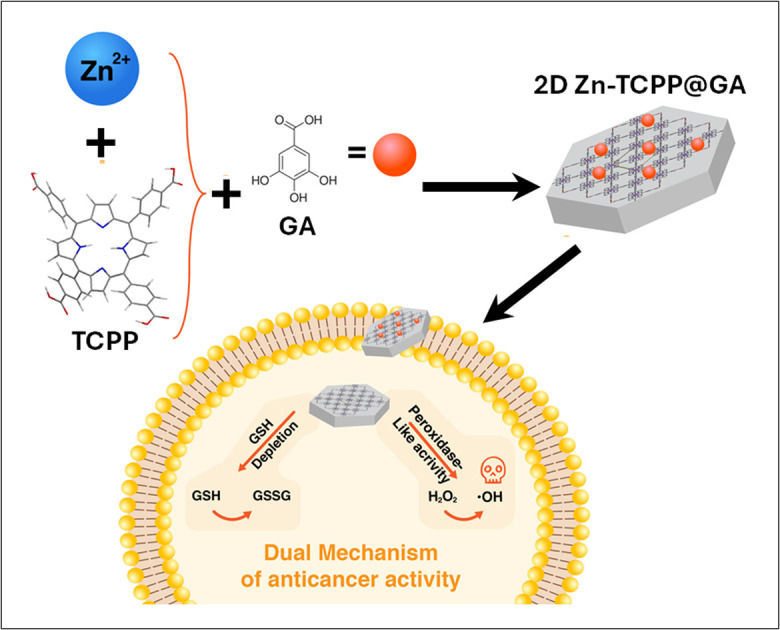

## Introduction

Nanomaterials exhibit considerable potential in cancer therapy due to their distinctive physicochemical properties. They can be used for a variety of purposes, such as drug delivery, bioimaging, gene therapy, immunotherapy and modulating the tumor microenvironment. This diversity of function makes nanomaterials a versatile platform for addressing the multifaceted nature of cancer treatment.^1^ In addition to their well-established applications, the use of nanomaterials as anti-cancer agents in photodynamic therapy (PDT) and chemodynamic therapy (CDT) is still a nascent research direction. PDT is a treatment that uses light-activated photosensitizing nanoagents to generate reactive oxygen species (ROS). While CDT takes advantage of the tumor tissue's elevated endogenous H_2_O_2_ levels and acidic pH, nanomaterials catalyze the conversion of H_2_O_2_ into highly reactive hydroxyl radicals (˙OH).^2^ These radicals induce oxidative stress, DNA damage, and protein inactivation, leading to cell death. Unlike PDT, CDT does not require external light, instead relying on the intrinsic properties of the tumor tissues, making it effective against deep-seated tumors. Moreover, CDT exhibits enhanced selectivity and minimized off-target damage, because the H_2_O_2_ level is elevated in tumor tissues compared to healthy tissues.^3^

Metal–organic frameworks (MOFs) have gained significant attention in recent years due to their potential applications in cancer research and therapy, including drug delivery, bioimaging, and as PDT and CDT anticancer agents. MOFs are hybrid porous materials composed of metal ions coordinated with organic ligands, forming a highly porous network with an extensive surface area. This structural characteristic enables MOFs to encapsulate a high payload of anticancer drugs. In addition to their high loading capacity, MOFs can be functionalized with tumor-targeting ligands, enabling site-specific delivery and minimizing off-target effects. Their responsiveness to stimuli and their ability to release drugs in a controlled manner can further enhance therapeutic efficacy.^4–7^ Furthermore, in contrast to many inorganic nanostructures, which often lack biodegradability, MOFs exhibit favorable biodegradability owing to the relative instability of their metal–ligand coordination bonds under physiological conditions.^8,9^

Notably, 2D MOFs represent a novel subclass that offers distinct advantages over conventional 3D MOFs, particularly in the realm of cancer diagnosis and therapy. Their planar architecture gives 2D MOFs a higher surface area-to-mass ratio compared to 3D MOFs, enabling significantly greater drug loading capacity and improved interaction with biological targets. This structural feature facilitates more efficient drug delivery. The organized molecular arrangement inherent to 2D MOFs also promotes stronger collective interactions, which are particularly beneficial in PDT and CDT, where the proximity and uniform distribution of active sites are critical for the efficient generation of ROS and tumor targeting. In a recent study, Wang *et al.* synthesized ultra-thin 2D MOF nanosheets and demonstrated their exceptional performance in eradicating tumor cells with high efficiency and selectivity.^10^ Building on previous work, the Cu-based 2D MOF nanosheets demonstrate potent chemodynamic activity by exploiting elevated intracellular GSH and H_2_O_2_ levels in the tumor microenvironment. Through GSH-triggered reduction of Cu^2+^ to Cu^+^, they catalyze Fenton-like reactions, enhancing ROS production, depleting GSH, and inducing tumor cell apoptosis predominantly *via* ferroptosis.^11^ In the same context, 2D Cu-bipyridine MOF [Cu(bpy)_2_(OTf)_2_] has shown improved CDT efficacy in colon cancer by rapidly consuming overexpressed hydrogen sulfide (H_2_S) to prevent ˙OH quenching, generating ultrasmall CuS to facilitate Fenton-like reactions, and enabling photothermal-enhanced CDT. Together, these findings underscore the versatility of 2D MOF nanosheets as effective and adaptable CDT agents for diverse cancer types.^12^

In the present study, a 2D MOF composed of zinc and a porphyrin ligand was selected for testing as an anticancer agent. Zinc-based complexes have emerged as an innovative alternative to conventional platinum-based chemotherapeutics, and zinc ions exhibit markedly lower systemic toxicity than metals such as iron, copper, and mercury, particularly at elevated doses. Zinc complexes have demonstrated the ability to catalyze DNA hydrolysis and cleavage in cancer cells, thereby contributing to their cytotoxic potential.^13^

Porphyrins, on the other hand, are well-established photosensitizers and have been widely utilized in PDT and CDT^7,14^ Their incorporation into MOF structures offers multiple therapeutic advantages. Notably, porphyrin-MOFs exhibit enhanced drug delivery capabilities and a high drug-loading capacity, as well as reduced aggregation, which prevents self-quenching in PDT and facilitates ROS diffusion.^15–17^ These MOFs also display intrinsic catalytic redox activity in the presence of H_2_O_2_*via* the redox-active metal center, which can cycle between oxidation states to produce hydroxyl radicals (˙OH), a key ROS in cancer cell apoptosis.^18–20^ Furthermore, porphyrins play a pivotal role in regulating the intracellular redox balance by targeting glutathione (GSH), a critical antioxidant tripeptide. Porphyrins can oxidise or bind GSH, thereby compromising the antioxidant defence systems of cancer cells.^19^ Concurrently, gallic acid (GA) has recently gained attention for its chemodynamic anti-cancer effects, which act through GSH depletion and ROS elevation to further amplify oxidative stress within tumor cells.^21,22^

**Fig. 1:**
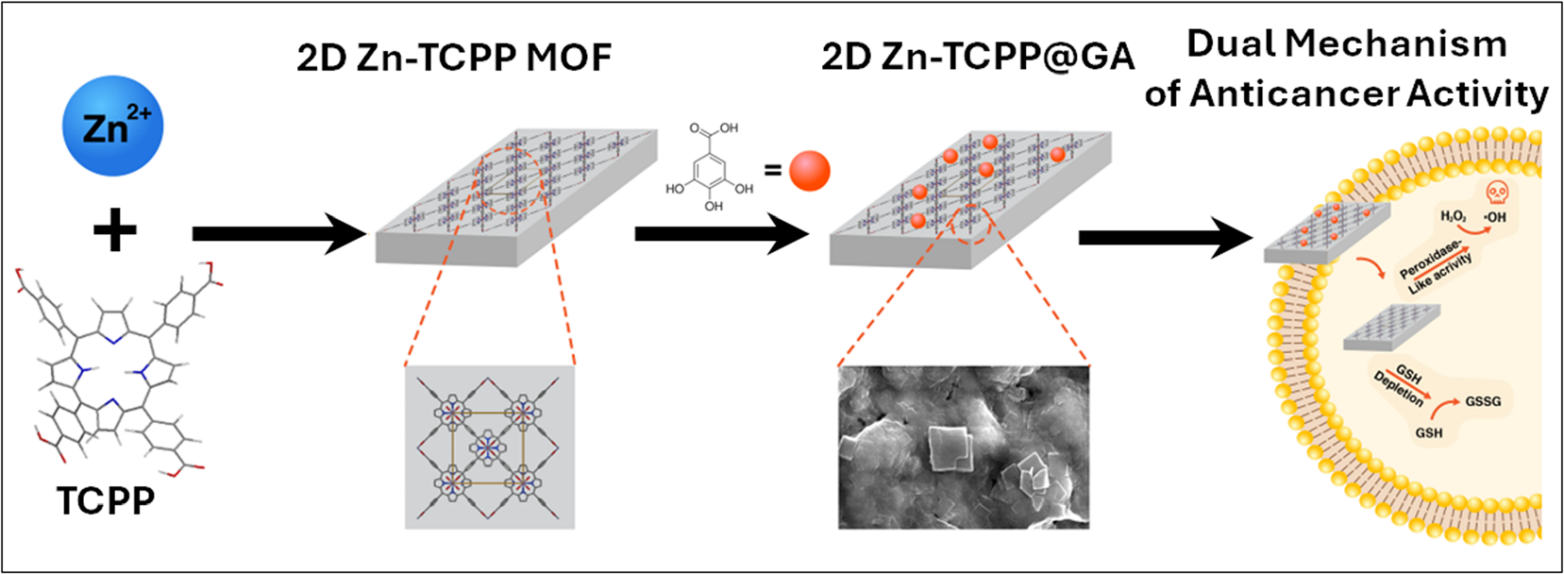
A schematic representation of the design and dual mechanism of action of Zn-TCPP@GA for the treatment of breast cancer.

In our current research, we are focusing on developing zinc–porphyrin MOF nanosystems co-loaded with GA as a multi-modal strategy for targeted cancer therapy. Our primary objective is to explore and improve the anticancer efficacy of these 2D MOF nanosheets by integrating GA, forming a hybrid material denoted as Zn-TCPP@GA. The 2D structure of Zn-TCPP MOFs has a high surface area, which facilitates efficient oxygen interaction and improves drug loading capacity. These nanosheets exhibit peroxidase-mimetic ROS generation activity, enabling the generation of ROS that contribute to the effective killing of cancer cells. Notably, the Zn-TCPP@GA system shows promising results in targeting breast cancer cells. Our findings highlight the potential of biodegradable 2D MOFs as safe and effective platforms for cancer therapies. Fig. 1 illustrates the design and dual mode of action of Zn-TCPP@GA composite MOF structure for the treatment of breast cancer.

## Materials and methods

### Materials

Zinc nitrate hexahydrate (Zn (NO_3_)_2_·6H_2_O, 99%), tetrakis(4-carboxyphenyl)porphyrin (TCPP), gallic acid (GA), and polyvinylpyrrolidone (PVP) were purchased from Sigma-Aldrich. The terephthaladehydic acid, pyrrole, all chemical reagents, and solvents used in experiments were analytically pure, purchased from Sigma-Aldrich. Methylene Blue (MB) and hydrogen peroxide (H_2_O_2_) were purchased from Fisher Scientific. Ultrapure water, provided by a Milli-Q purified system, was used in all preparations.

### Methods

#### Meso-tetrakis(4-carboxyphenyl) porphyrin (TCPP) synthesis

Synthesis of TCPP linker was performed by following the method mentioned elsewhere.^23^ Refluxing equimolar portions of pyrrole (0.2 mL, 0.193 g, 2.88 mmol) and terephthaladehydic acid (0.3380 g, 2.25 mmol) was conducted with an excess of propionic acid (100 mL, 99.3 g, 1.34 mol). The solution was kept under reflux for two hours and the reaction being observed *via* thin layer chromatography (TLC). After the reaction is finished, the solution is left to cool down. The solution is then filtered and rinsed with methanol. After being allowed to air dry, the precipitate is collected as a dark purple powder, resulting in a 12.53% yield.

#### Synthesis of 2D Zn-TCPP-MOF

Synthesis Zn-TCPP-MOF was reported in the original ref. 16 have been performed with minor modifications. Typically, 2.0 mg TCPP was firstly dissolved in dimethylformamide (DMF) (10 mL), followed by ultrasonic treatment for 1 min. 8.0 mg of Zn(NO_3_)_2_·6H_2_O was added to 5 mL of ethanol then both solutions mixed, ultrasonication for 2 min to ensure solid fully dissolved. Then, 2 mg PVP was added to the mixture. Finally, the mixed system was kept under heating and stirring 120 °C for 2 hours. After the system was cooled down to room temperature naturally, a glittery like purple to red precipitate was formed and separated by centrifuging at 6000 rpm for 10 minutes. Finally, the obtained product was washed three times with ethanol, dried at 60 °C under vacuum for 24 h. The chemical structure of Zn-TCPP is demonstrated in Fig. S1a.

### Characterization of Zn-TCPP-MOF

The morphological characterizations were conducted using SEM. SEM analysis was carried out with a field emission microscopy instrument (FEI Inspect F50, USA). Additionally, the elemental composition of Zn-TCPP-MOF was determined *via* Energy-Dispersive X-ray Spectroscopy (EDS) during the SEM analysis. X-ray Diffraction (XRD) for the MOF was conducted using Rigaku MiniFlex XRD system. TGA (Thermal Gravimetric Analysis) and the BET (Brunaner–Emmett–Teller) analysis were done using METTLER TOLEDO and micrometrics instrument respectively. Adsorption–desorption experiment at −195.850 °C, N_2_ as medium and equilibrium time interval of 10 s. The adsorption data was measured at the relative pressure (*P*/*P*_0_) between 0.05 and 0.30. While the Fourier transform infrared (FTIR) spectrum was performed on Nexus instrument. Fluorescence and absorbance were measured in Duetta instrument from Horiba company.

### GA loading and surface modification of Zn-TCPP

The structure of GA is demonstrated in Fig. S1b. Loading of GA was conducted followed the procedure mentioned before with minor modifications.^24^ An amount of 0.001 mg of GA in 10 mL water were stirred for one hour till complete dissolution of GA. Then, Zn-TCPP nanosheets (0.010 g) were added to this solution. The mixture was kept at pH 8.0 using a PBS buffer solution and was stirred for 24 h. The products were separated by centrifugation then washed by ethanol and then water five times to remove the un-loaded GA and were suspended in ethanol for future use. For Encapsulation Efficacy % (EE%) calculations, the resultant supernatant was kept for the absorbance measurement at 270 nm of gallic acid using a UV-visible spectrometer, using the equation below:% EE = [amount of GA in filtrate/amount of GA added during preparation] × 100

### Characterization of Zn-TCPP@GA nanosheets

SEM-EDX analysis of the Zn-TCPP@GA nanosheets was carried out to study the morphology and elemental composition of the proposed anticancer candidate. The UV-visible spectrum was measured to ensure the presence of GA. In addition, the phase composition of the Zn-TCPP@GA nanosheets was investigated using XRD, FTIR and TGA techniques.

The evaluation of the drug release kinetics, the *in silico* evaluation of the affinity of gallic acid towards Zn-TCPP MOF structure and the details of the extracellular detection of the hydroxy radicals as well as the details of the procedures used for the cytotoxicity of the GA-loaded Zn-TCPP are shown in the SI section.

## Results and discussion

In our work, we synthesized Zn-TCPP 2D nanosheets using the solvothermal method, with PVP acting as a surfactant and modulator for the reaction. The Zn-TCPP MOF nanosheets contain numerous carboxyl groups on their surface, with each ligand molecule having four of these groups. This abundance of carboxyl groups offers a numerous sites for further potential modification reactions.^25^

**Fig. 2:**
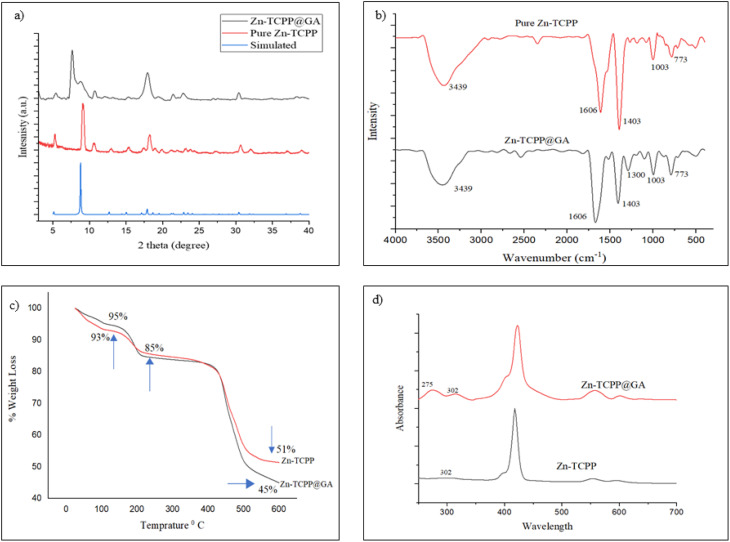
(a) XRD diffractogram for Zn-TCPP pure MOF, Zn-TCPP@GA and simulated MOF, (b) FTIR spectra for Zn-TCPP and Zn-TCPP@GA with highlighted band specific for GA, (c) TGA analysis of both Zn-TCPP and Zn-TCPP@GA, (d) UV-vis spectra of pure MOF and MOF@GA.

Regarding the crystalline structure of Zn-TCPP MOFs, previous studies have shown that the 2D framework is formed by the coordination four zinc paddlewheel nodes of the formula Zn_2_(COO)_4_ with tetra(4-carboxyphenyl)porphyrin (TCPP) ligands. These planar sheets then stack together in a layered structure with an AB stacking arrangement.^26^ The crystallinity of the synthesized Zn-TCPP MOF was confirmed by X-ray diffraction (XRD) analysis. The XRD pattern exhibits characteristic diffraction peaks at 2*θ* values of 5.7°, 7.6°, 9.2°, 18.3°, 21.0°, and 30.0° (Fig. 2a), which are in close agreement with the simulated diffraction pattern of the ideal Zn-TCPP MOF structure (Fig. 2a). These results confirm the successful formation of a well-defined, crystalline 2D MOF architecture.

The XRD patterns presented in Fig. 2a compare the diffraction patterns of both the pure Zn-TCPP MOF and the GA-functionalized composite (Zn-TCPP@GA). The XRD patterns reveal a notable similarity in the characteristic peaks of the two samples, indicating that the overall crystalline framework of the Zn-TCPP MOF is largely retained following GA incorporation. Moreover, the peak positions align well with previously reported data for Zn-TCPP structures.^26^ However, a reduction in the intensity of the peak near 9°, corresponding to the (011) lattice plane, suggests a slight reduction in crystallinity. Additionally, the broadening and slight shift of peaks around 9° and 18° toward lower 2*θ* values indicate a reduction in layer stacking and an increased interlayer spacing, consistent with previously reported changes upon molecules loading.^27^ A new diffraction peak at 7.6°, absent in the pure Zn-TCPP MOF, emerges in the Zn-TCPP@GA sample and is attributed to the potential intercalation of GA molecules into the MOF layers,^28^ as will be further confirmed in a later section. The reduction in crystallinity of Zn-TCPP@GA is likely due to the immobilization of the organic moiety (GA), while the lower peak intensities further confirm that a diffusion process has occurred within the structure of the parent MOF.^27^ These features suggest the successful loading of GA into the MOF framework, while maintaining the structural stability of the MOF framework (Fig. S1c–e).

FTIR spectroscopy was conducted for both Zn-TCPP and Zn-TCPP@GA, with the resulting spectra displayed in Fig. 2b. The FTIR spectrum of the as-prepared Zn-TCPP MOF shows two characteristic bands near 1606 cm^−1^ and 1403 cm^−1^, which can be attributed to the O–C–O stretching vibrations coordinated to Zn^2+^ ions, indicating the formation of coordination bonds between the carboxyl groups of the TCPP linker and zinc centers.^29^ A broad band around 3437 cm^−1^ corresponds to the O–H stretching vibration of carboxylic acid groups.^30^ The presence of a peak at 498 cm^−1^ is assigned to the Zn–O vibrational mode, associated with the Zn_2_(COO)_4_ paddlewheel units in the MOF structure, confirming successful coordination between Zn^2+^ ions and the porphyrin linker. Additionally, a peak at 720 cm^−1^ is attributed to the in-plane bending vibration of methylene C–H bonds, while a distinct band at 1001 cm^−1^ corresponds to the Zn–N stretching mode, further confirming the successful assembly of the MOF framework.^31^ In comparison, the FTIR spectrum of Zn-TCPP@GA exhibits similar characteristic bands, indicating the preservation of the core MOF structure. Notably, an additional band appears around 1300 cm^−1^, which is assigned to the C–O stretching vibration of the phenolic group in GA, indicating a successful GA incorporation within the Zn-TCPP MOF structure.^32^

The TGA curve shown in Fig. 2c reveals a clear difference in the total weight loss between the pure Zn-TCPP MOF and the GA-functionalized Zn-TCPP@GA structures. Both samples exhibit three distinct thermal events, occurring at approximately 140 °C, 190 °C, and 430 °C. These thermal events correspond, respectively, to the removal of weakly adsorbed solvent molecules, the initial degradation of the MOF framework, and the complete decomposition and breakdown of the hierarchical structure of both MOFs. The pure Zn-TCPP MOF displayed an overall weight loss of 49%, whereas the Zn-TCPP@GA MOF exhibited a slightly higher total weight loss of 51%. This increase is primarily attributed to the loss of GA component integrated into the Zn-TCPP@GA structure. These findings were further confirmed by the DTG analysis of these samples, as shown in Fig. S2. The increased weight loss of the Zn-TCPP@GA structure is evident at 190 °C and 430 °C. The conjugation efficiency of GA into Zn-TCPP was calculated to be approximately 6%, indicating that each 1 mg of Zn-TCPP MOF can incorporate up to 60 µg of GA.

The UV-vis absorption spectra of both Zn-TCPP and Zn-TCPP@GA structures are presented in Fig. 2d. The absorption band observed at 309 nm confirms that Zn^2+^ ions have successfully coordinated with the porphyrin core, indicating metalation through interaction with the four pyrrole nitrogen atoms.^8^ Additionally, the absorption band around 270 nm, characteristic of GA, is clearly visible in the Zn-TCPP@GA spectrum, suggesting successful incorporation of GA into the MOF structure. Furthermore, the main band of the TCPP ligand, initially observed around 420 nm, exhibits a slight red shift following MOF formation. This shift is attributed to ligand-to-metal charge transfer, which results in absorption at longer wavelengths, consistent with previous observations for metalated porphyrin systems.^25^

**Fig. 3:**
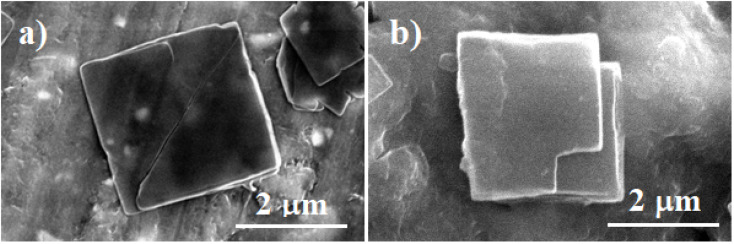
SEM images for Zn-TCPP pure MOF (a) and Zn-TCPP@GA (b).

The SEM micrographs of the pure Zn-TCPP MOF structure show its multilayered stacked 2D sheets, consistent with what has been reported in previous studies (Fig. 3a). Upon modification of the Zn-TCPP with GA, no noticeable change in the morphology was found (Fig. 3b). In both cases, the average size of the 2D sheets was around 2 µm. The elemental analysis of the as-prepared Zn-TCPP MOFs (Fig. S3a) showed the presence of Zn, N, C, and O with atomic percentages of 1.69, 4.98, 32,11, and 8.7 respectively. On the other hand, the elemental analysis of the GA-modified Zn-TCPP (Fig. S3b) showed a noticeable increase in the atomic percentages of C and O to 42.48 and 24.96%, respectively. The difference in the atomic concentrations of the C and O in the GA-modified Zn-TCPP could be related to the presence of GA in the structure. Fig. S4 shows the N_2_-asorption hysteresis of the as-prepared Zn-TCPP and the GA-modified Zn-TCPP. The pristine Zn-TCPP exhibits a typical type-IV isotherm with a pronounced hysteresis loop, confirming its mesoporous nature and accessible internal pore network (Fig. S4a). After GA loading, the adsorption capacity at low relative pressure is significantly suppressed, indicating that the internal pores are largely occupied or blocked by GA molecules (Fig. S4b). In contrast, a sharp increase in adsorption near to *P*/*P*_0_ → 1 is observed, which is mainly attributed to nitrogen condensation in interparticle voids. The substantial widening of the hysteresis loop after GA loading further confirms pore blocking and restricted desorption, providing strong evidence for successful incorporation of GA within the MOF framework. Compared with a BET surface area of 870 m^2^ g^−1^ for the as-prepared Zn-TCPP, the loading of GA into/onto the Zn-TCPP structure resulted in a decrease in the BET surface area 445 m^2^ g^−1^. The decrease in the BET surface area is attributed to the partial blocking of the Zn-TCPP porosity due to the immobilization of GA into/onto the Zn-TCPP 2D structure.

Fig. S5 shows the pore size distribution of the as-prepared Zn-TCPP and the GA-modified Zn-TCPP. The pristine Zn-TCPP shows a high pore size distribution intensity reaching approximately 0.07 cm^3^ nm^−1^ g^−1^ in the small pore region, with a broad distribution extending from the microporous/low-mesoporous range up to about 140 nm, confirming its highly porous and accessible framework. After GA loading, the pore size distribution intensity dramatically decreases to the order of 10^−4^ cm^3^ nm^−1^ g^−1^, representing a reduction of nearly three orders of magnitude compared to the pristine sample. In addition, the characteristic contributions from small pores almost completely disappear, indicating that these pores are largely occupied or blocked by GA molecules. The remaining distribution shifts toward much larger apparent pore widths (above 100 nm), which are mainly attributed to interparticle voids rather than internal MOF porosity. This pronounced decrease in pore volume, disappearance of small pore contributions, and shift toward larger pore sizes provide strong quantitative evidence that GA is successfully loaded into the internal pore channels of Zn-TCPP, effectively occupying and blocking the original porous network of the MOF.

### Gallic acid binding and stabilization

**Fig. 4:**
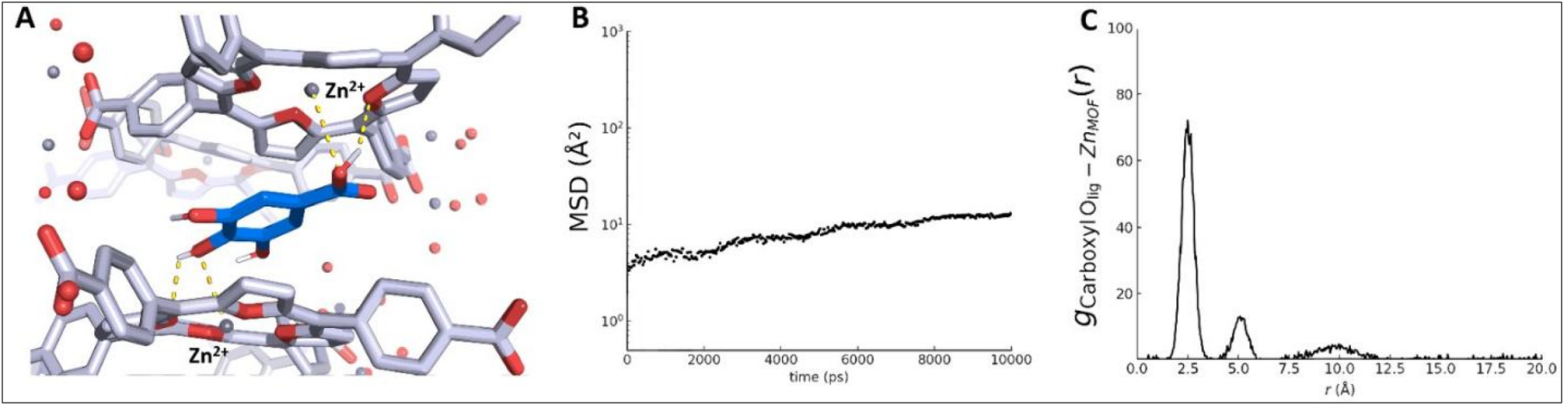
(A) Representative MD snapshot showing gallic acid (GA; blue) stably confined between two porphyrinic layers of the Zn-TCPP framework. GA exhibits π–π-like aromatic packing (centroid distance ∼3.7–4.0 Å; interplanar separation ∼3.4 Å), while the deprotonated carboxylate forms short Zn–O contacts (∼2.1–2.3 Å) with Zn^2+^ centers (gray spheres) above and below; the para-hydroxyl forms hydrogen bonds (yellow dashed lines) with nearby framework oxygen atoms (H⋯O ∼1.8 Å; occupancy >50%). (B) Mean squared displacement (MSD) of GA within the rigid Zn-TCPP MOF over a 10 ns analysis window, indicating restricted mobility. (C) RDF between GA carboxylate oxygens and framework Zn centers, with a primary peak at ∼2.5–2.7 Å (strong GA–Zn interaction) and a secondary peak near ∼5.2 Å (binding involving two Zn centers), consistent with localized confinement. The absence of long-range peaks beyond 7 Å reflects the localized confinement of gallic acid within the pore environment.

The MD trajectory reveals that gallic acid quickly localizes to a specific binding site, where it remains stably sandwiched between two TCPP porphyrin layers of the framework throughout the 100 ns simulation (Fig. 4A). The aromatic ring of gallic acid aligns roughly parallel to the porphyrin linkers above and below it, indicating face-to-face π–π stacking interactions. This type of stacking of aromatic guest molecules between aromatic MOF linkers has been observed in similar host–guest systems (*e.g.* perylene guests π-stacked between benzene linkers in a MOF).^33^ In our system, the centroid-to-centroid distance between the gallic acid phenyl ring and a porphyrin phenyl ring is on the order of ∼3.7–4.0 Å, with an interplanar separation of ∼3.4 Å, which is consistent with typical π–π stacking geometries.^34^ This π-stacking interaction significantly stabilizes the guest molecule in the pore. In addition, gallic acid's polar functional groups form strong site-specific interactions with the framework. Notably, the deprotonated carboxylate group of gallic acid is oriented towards two Zn^2+^ centers directly above and below the guest. Each carboxylate oxygen lies within ∼2.1–2.3 Å of a framework Zn atom (Fig. 4A), suggesting a bidentate coordination or strong electrostatic attraction to these open metal sites. Such coordinatively unsaturated Zn sites (created upon activation by removal of bound solvent) are known to bind guest oxygen atoms strongly,^35^ and here they serve to anchor the carboxylate of gallic acid on both sides. Furthermore, the para-hydroxyl group of gallic acid participates in hydrogen bonding with the framework. Throughout the simulation, this hydroxyl forms a hydrogen bond (occupancy >50%) with a nearby oxygen (likely a carboxylate oxygen of a TCPP linker or a Zn–OH group) at a distance of ∼1.8 Å. The gallic acid molecule is therefore immobilized by multiple host–guest interactions, including π–π stacking between aromatic rings, coordinative bonding to Zn nodes, and hydrogen bonding to framework oxygens. These synergistic interactions result in a high-affinity binding site. Indeed, once gallic acid diffuses into this site in the early stage of the simulation, it remains there without escaping, indicating the energy barriers for desorption or relocation are quite high. This behavior is reminiscent of other MOF-guest systems where strong hydrogen bonding or π–π interactions dominate the adsorption site preference.^36^ In our case, both types of interactions cooperate to lock the guest in place.

The time evolution of the mean squared displacement (MSD) of gallic acid within the Zn-TCPP framework is presented in Fig. 4B. Over the course of the 10 ns simulation window, the MSD remains below 20 Å^2^, with a gradual increase that begins to plateau after approximately 6 ns. The overall trend is sub-linear, reflecting a transition from initial translational freedom to restricted, localized motion within a confined binding site.

Notably, the MSD starts around 3–5 Å^2^ and increases modestly to ∼11 Å^2^ by the end of the simulation. This behavior indicates that gallic acid undergoes limited spatial displacement, characteristic of caged dynamics within a high-affinity binding pocket. The lack of a sustained linear regime in the MSD curve implies that gallic acid is not undergoing free diffusion through the pore system but is instead restricted to vibrational and re-orientational motions within a fixed region. This confinement is consistent with its strong binding interactions – particularly the bidentate coordination to Zn centers and persistent hydrogen bonding with the framework carboxylate and porphyrin groups.^37^ These findings underscore the capability of the Zn-TCPP framework to act as a high-affinity, low-permeability host for gallic acid, reinforcing its potential use in applications that require controlled retention or release of phenolic bioactive molecules.

### Gallic acid-Zn-TCPP structural correlations (RDF analysis)

The radial distribution function (RDF) between the carboxylate oxygen atoms of gallic acid and the Zn centers of the Zn-TCPP framework is shown in Fig. 4C. The RDF profile exhibits a sharp and prominent peak centered at approximately 2.5 Å, reaching a maximum value exceeding 70, which is indicative of a highly structured and specific interaction. This intense and well-defined peak corresponds to a strong coordinative or electrostatic interaction between the deprotonated carboxylate group of gallic acid and the Zn^2+^ nodes in the framework.

A second, broader peak is observed near 5.2 Å, suggesting a secondary coordination shell or the spatial relationship of the second oxygen atom from the carboxylate group with a neighboring Zn site. The presence of these two peaks supports a symmetric bidentate-like binding geometry where the carboxylate is stabilized by two Zn atoms located above and below the gallic acid molecule, consistent with the sandwiched configuration visualized in Fig. 4A.

Beyond 7 Å, the RDF decays toward unity with minor fluctuations, indicating that there are no other strong or directional interactions between the carboxylate oxygen and distant Zn centers. The minimal long-range structure and the absence of additional pronounced peaks reflect the localized nature of the coordination environment.

Overall, the RDF data provide strong quantitative evidence of site-specific coordination between gallic acid and Zn centers in the MOF. The exceptionally high intensity of the first RDF peak further underscores the stability and rigidity of the host-guest complex, validating the structural confinement observed in the MD trajectory.

MD identifies a high-affinity binding pocket in which GA is π-stacked between porphyrinic linkers and coordinated *via* its carboxylate to Zn sites. This host-guest geometry explains the observed XRD evolution: a new low-angle reflection at 2*θ* ≈ 7.6° (*d* ≈ 11.6 Å) and a slight shift/broadening of peaks near 9°/18°, indicative of interlayer expansion and diminished stacking coherence. The strong Ocarb–Zn coordination peak in the RDF (2.5–2.7 Å) provides an atomistic basis for the emergence and stability of this expanded periodicity.

### Uptake capacity

Using GCMC simulations, the maximum gallic acid uptake capacity of Zn-TCPP was estimated under idealized dry-loading conditions. Assuming one gallic acid molecule per porphyrinic cavity a stoichiometry supported by GCMC equilibrium sampling, the gravimetric uptake was calculated the previously mentioned equation in the methods.

This yielded an uptake value of approximately 265 mg of gallic acid per gram of Zn-TCPP MOF. This estimate reflects the intrinsic adsorption potential of the framework in the absence of solvent competition. It is anticipated that in aqueous or physiological conditions, the effective uptake may be modestly reduced due to competitive binding by water molecules at the Zn^2+^ sites. Nevertheless, the combination of strong π–π stacking, bidentate coordination, and hydrogen bonding, as demonstrated in our MD simulations, suggests that Zn-TCPP remains a promising scaffold for high-affinity phenolic compound encapsulation. GCMC predicts an upper-bound loading in the idealized dry limit (∼26.5 wt%). Experimentally, TGA quantifies ∼6 wt% under solvated conditions, consistent with competitive adsorption by water/ions and kinetic/heterogeneity constraints. The agreement in trends*-*strong affinity sites and stable confinement from MD/RDF, alongside XRD evidence of interlayer expansion-supports successful GA incorporation even though the realized loading is below the ideal maximum.

### GA encapsulation efficiency and *in vitro* GA, TCPP and Zn ions release studies

The encapsulation efficiency (%EE) of GA was determined using UV-vis spectroscopy and was found to be 25%. This relatively high percentage can be attributed to the large surface area and high chemical functionality of the Zn-TCPP MOF, which facilitates hydrogen bonding and electrostatic interactions between the O–H and COOH groups of GA and the TCPP ligands and Zn^2+^ ions within the MOF framework. It has been demonstrated that GA, TCPP, and Zn^2+^ ions each possess intrinsic anticancer properties. So, in the present study, the release kinetics of GA, TCPP, and Zn^2+^ ions from the Zn-TCPP@GA composite were examined to assess their contribution to anticancer activity. The release profiles of GA and TCPP were analyzed through UV-vis spectroscopy using various kinetic models to evaluate the release mechanisms, including zero-order, first-order, Higuchi, Hixson, and Korsmeyer-Peppas models. These models are schematically illustrated in Fig. S6, and the corresponding *R*^2^ values are shown in Table 1.

**Table 1:** *R* ^2^ (The proportion of the variance in the dependent drug release variable) values of each model

*R* ^2^ values	Zero order	First order	Higuchi	Korsmeyer–Peppas	Hixon
GA	0.85	0.86	0.95	0.86	0.86
TCPP	0.91	0.94	0.99	0.92	0.95

Our findings indicate that the release kinetics of GA and TCPP from the Zn-TCPP@GA MOF structure are best described by the Higuchi model. This suggests that GA is not only adsorbed on the surface but also incorporated within the pores and interlayer spaces of the Zn-TCPP MOF. Upon exposure to the release medium, the Zn-TCPP@GA structure undergoes surface degradation, which facilitates the diffusion of GA, TCPP, and Zn^2+^ ions into the surrounding environment. In general, the Higuchi model is commonly applied to describe drug release from polymeric or porous systems, taking into account mechanisms such as diffusion through the matrix, polymer degradation, and erosion.^38^

**Fig. 5:**
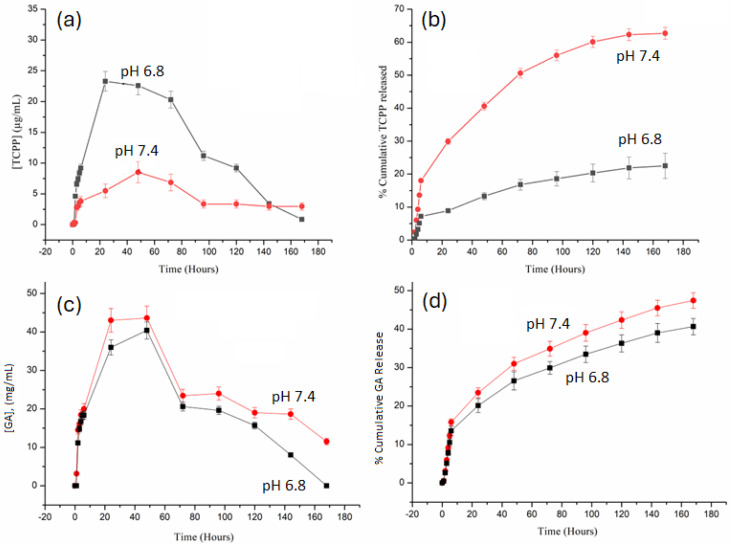
Cumulative profile showing the release of TCPP and GA as a function of soaking time in PBS at different pH values, shown as changes in the concentration of TCPP (a), its cumulative release (b) and changes in the concentration of GA (c) and its cumulative release (d).

Additionally, the release profiles of TCPP and GA from the Zn-TCPP@GA framework at different pH conditions are illustrated in Fig. 5a–d. Both cargos exhibit a biphasic release pattern, with an initial burst phase within the first 24 hours followed by a slower, diffusion-controlled stage. As shown in Fig. 5a, the cumulative release of TCPP reaches approximately 63% at pH 6.8 after 160 hours, compared with only ∼22% at pH 7.4, indicating pronounced pH-responsive behavior. The corresponding concentration–time profile in Fig. 5b shows an early peak in TCPP concentration (∼25 µg mL^−1^ at pH 6.8 *vs.* ∼8 µg mL^−1^ at pH 7.4), followed by a gradual decline due to equilibration or re-adsorption of released molecules.

Similarly, Fig. 5c and d display the release kinetics of GA, which follow the same general trend but with significantly lower total release than TCPP at both pH values. This difference arises from GA's stronger Zn–O(carboxylate) coordination and extensive hydrogen bonding with the framework, as revealed by molecular-dynamics simulations and RDF analysis, which predict persistent Ocarb–Zn interactions around 2.5–2.7 Å. In contrast, TCPP is primarily stabilized by π–π stacking, which is more easily disrupted under acidic conditions.

At physiological pH 7.4, the Zn-TCPP@GA composite demonstrates excellent structural stability, exhibiting minimal cargo leakage and correlating well with XRD data showing preserved crystallinity and only slight peak broadening, as well as TGA results confirming high thermal robustness. In mildly acidic media (pH 6.8), partial protonation of carboxylate groups weakens Zn–O coordination and expands the interlayer spacing, thereby facilitating diffusion and release. The results in Fig. 5a–d confirm that Zn-TCPP@GA functions as a stimuli-responsive nanocarrier, maintaining integrity under physiological conditions while enabling accelerated and selective release of both TCPP and GA in acidic tumor-like environments; a desirable feature for controlled photodynamic and chemodynamic therapy applications.

Fig. 5a shows that Zn^2+^ release from the Zn-TCPP framework is strongly pH-dependent. At pH 6.8, the Zn^2+^ concentration rises rapidly within the first 24 hours, reaching values above 12 ppm, while at pH 7.4 the release remains much lower, peaking around 4 ppm. This behavior indicates that acidic conditions promote partial cleavage of Zn–carboxylate coordination bonds, leading to accelerated dissolution of Zn nodes. In contrast, the limited Zn^2+^ release at pH 7.4 confirms the structural stability of the framework under physiological conditions.

The cumulative profiles in Fig. 5b further emphasize this effect, with nearly 70% Zn^2+^ release at pH 6.8 compared to only ∼47% at pH 7.4 over 168 hours. The enhanced Zn^2+^ liberation in mildly acidic media aligns with the behavior observed for TCPP and GA release and reflects proton-induced weakening of the framework. Overall, these results demonstrate that Zn-TCPP exhibits desirable pH-responsive degradation, favoring enhanced release in acidic, tumor-like environments while remaining stable at physiological pH.

### Extracellular detection of hydroxyl radical (˙OH) radical release

The highly cytotoxic hydroxyl radical (˙OH) has the ability to inflict damage to DNA, proteins, lipids, and other cellular components by inducing oxidative stress, which is a state characterized by an imbalance between the generation of ROS and the cell's antioxidant defense mechanisms. In this study, the developed nanostructures composed of Zn^2+^ ions, TCPP, and GA were engineered to exhibit peroxidase-mimetic ROS generation mechanism. Under the mildly acidic conditions typical of the tumor microenvironment, these nanostructures effectively catalyze the decomposition of H_2_O_2_ into highly reactive hydroxyl radicals. The generation of ˙OH radicals leads to oxidative damage within cancer cells, and ultimately inducing apoptosis or necrosis. This mechanism amplifies the therapeutic potential of the nanostructures by exploiting the elevated H_2_O_2_ levels often present in tumor tissues, offering a selective and efficient cancer treatment strategy.^17^

A more credible ROS generating facilitated by Zn-based nanostructures might involve Zn(ii)-coordinated species enhancing H_2_O_2_ decomposition *via* interactions with other catalytic components, such as TCPP or GA, which can mediate electron transfer.^19^

To evaluate the intrinsic catalytic activity of Zn-TCPP@GA and its ability to generate ˙OH radicals, a methylene blue (MB) degradation assay was used. MB undergoes degradation in the presence of ˙OH, which can be precisely tracked by fluorescence measurements. As shown in Fig. 7a, a noticeable decrease in MB absorbance was observed when MB was incubated with H_2_O_2_ and varying concentrations of Zn-TCPP@GA for 30 minutes in a NaHCO_3_/CO_2_ buffer system. In contrast, no significant change in absorbance occurred under the same conditions in aqueous solution, and H_2_O_2_ alone exhibited minimal effect on MB degradation in the buffer. These results suggest that the presence of both Zn-TCPP@GA and H_2_O_2_, in combination with bicarbonate ions, is vital for the efficient generation of ˙OH radicals. The rapid degradation of MB under these conditions indicates that the Zn-TCPP@GA nanostructure can mimic peroxidase enzyme activity and catalyze ˙OH production in physiologically relevant environments.

**Fig. 6:**
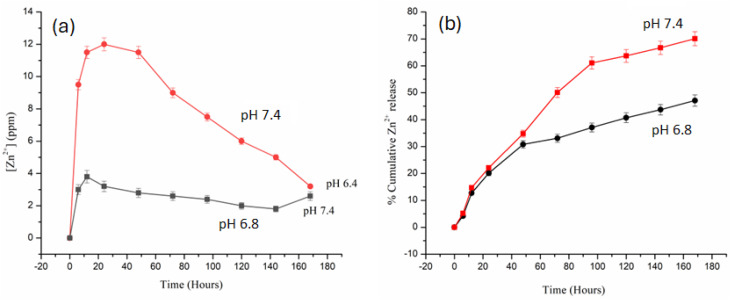
Zn ions release from Zn-TCPP@GA nanosheets at different pH values, shown as changes in the concentrations of Zn^2+^ ions (a) and its cumulative release (b).

To further confirm ˙OH generation, terephthalic acid was employed as a fluorescent probe. Terephthalic acid is a non-fluorescent molecule that reacts with ˙OH to form 2-hydroxyterephthalic acid, which emits strong fluorescence at 440 nm. As shown in Fig. 6b, increasing concentrations of Zn-TCPP@GA at a constant H_2_O_2_ level resulted in a proportional increase in 2-hydroxyterephthalic acid fluorescence, providing additional evidence for the catalytic production of ˙OH by Zn-TCPP@GA in the presence of H_2_O_2_.^39^

**Fig. 7:**
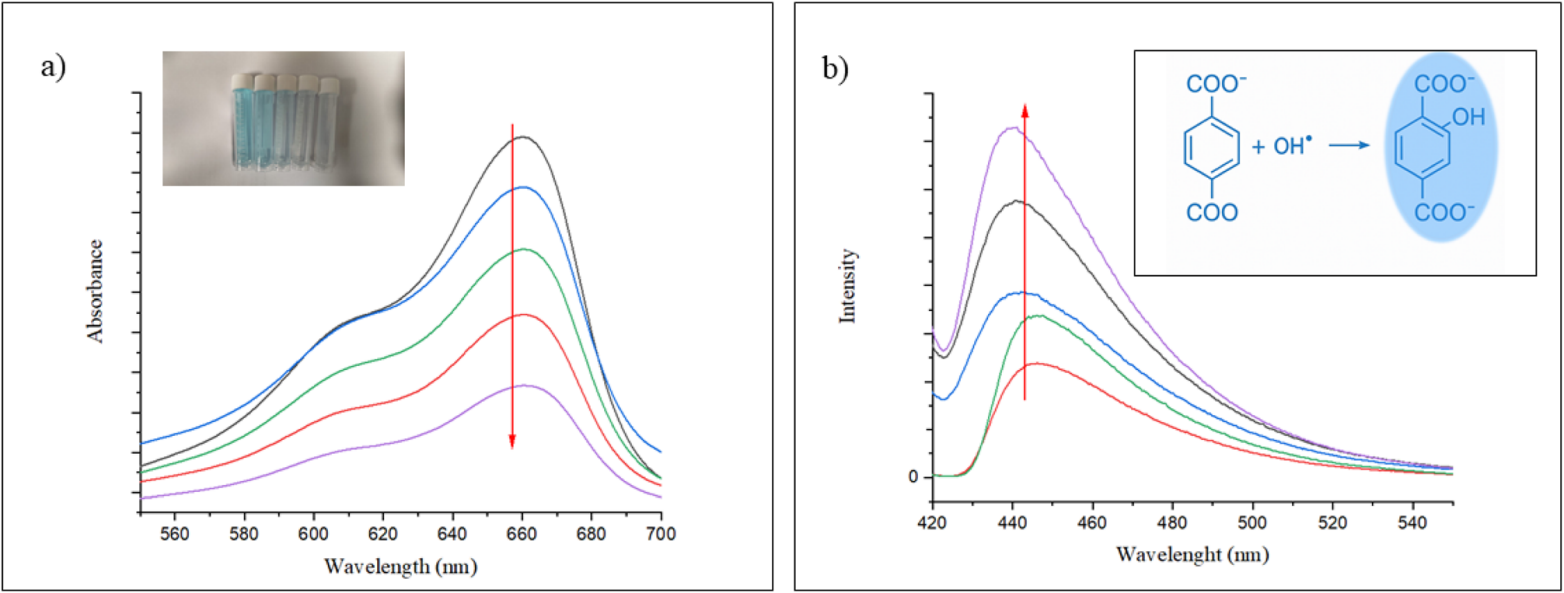
(a) MB absorbance fades out as the conc of Zn-TCPP@GA increases, (b) TA fluorescence increases with the increase in Zn-TCPP@GA concentration.

### Cytotoxicity assay

**Fig. 8:**
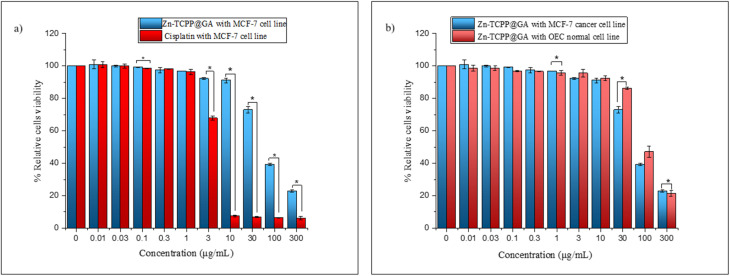
(a) MTT assay results demonstrate relative viability of cells treated with different concentrations of Zn-TCPP@GA and cisplatin against cancer MCF-7 cell line, (b) the action of Zn-TCPP@GA toward cancer MCF-7 and normal OEC cell lines.

Cytotoxicity assessment was performed using the MTT assay on MCF-7 breast cancer cells and OEC normal oral epithelial cells to evaluate the *in vitro* antitumor efficacy and biocompatibility of the Zn-TCPP@GA nanostructure. In addition to quantitative analysis, cell proliferation and morphological changes were monitored using an inverted phase-contrast microscope. The half-maximal inhibitory concentration (IC_50_) values for Zn-TCPP@GA were determined and compared with those of cisplatin, a clinically established metal-based chemotherapeutic agent, against MCF-7 cells. The use of MCF-7 cells enabled a focused evaluation of the anticancer efficacy of Zn-TCPP@GA and its comparative therapeutic potential relative to conventional treatments. Fig. 8 demonstrates that all treatment groups exhibited dose-dependent cytotoxicity, underscoring the effectiveness of Zn-TCPP@GA as a potential anticancer agent.

The IC_50_ values confirm this dose-responsive behavior following 48 hours of incubation with either Zn-TCPP@GA or cisplatin (Fig. 8a). Zn-TCPP@GA exhibited an IC_50_ value of 75.04 µg mL^−1^ against MCF-7 cells, whereas cisplatin showed significantly higher potency with an IC_50_ of 3.59 µg mL^−1^, as expected. Interestingly, the IC_50_ value of Zn-TCPP@GA was higher than those reported for other zinc-based nanostructures,^40,41^ suggesting moderate cytotoxicity. Furthermore, the differential cytotoxicity between cancerous and normal cell lines was estimated (Fig. 8b). Zn-TCPP@GA exhibited a higher IC_50_ value of 82.78 µg mL^−1^ in OEC cells, compared to 75.04 µg mL^−1^ in MCF-7 cells. This indicates lower toxicity towards healthy tissues, suggesting a favorable safety profile.

The cytotoxic effects of the tested drug were statistically compared to those of the standard treatment (cisplatin) at various concentrations using a Student's *t*-test (Fig. 8a). The results, expressed as the mean ± standard deviation (SD), revealed significant differences at specific concentrations. At lower concentrations (0–1 µM), there was no statistically significant difference in cytotoxicity between Zn-TCPP@GA and cisplatin (*p* > 0.05), suggesting comparable effects. However, at 0.1 µM, Zn-TCPP@GA exhibited a slightly increased cytotoxic effect than cisplatin (*t* = 3.504, *p* = 0.025). A substantial difference in cytotoxicity emerged at higher concentrations (≥3 µM). Zn-TCPP@GA exhibited a significantly higher cytotoxic effect compared to cisplatin, with *p*-values consistently below 0.001 across these concentrations. At 3 µM, Zn-TCPP@GA maintained 92.27% cell viability, in contrast to 67.83% for cisplatin (*t* = 24.589, *p* < 0.001), indicating a notable improvement in therapeutic efficacy. This trend became even more pronounced at 10, 30, 100, and 300 µM, where Zn-TCPP@GA maintained significantly higher cell viability compared to cisplatin, demonstrating its enhanced potency at elevated doses.

Further, the cytotoxic effects were evaluated in both normal and cancerous cells (Fig. 8b). At lower concentrations (0–1 µM), no statistically significant differences were observed between the two cell types (*p* > 0.05), indicating a similar cytotoxic response. However, at a concentration of 0.1 µM, the MCF-7 cancer cells demonstrated significantly greater sensitivity to Zn-TCPP@GA than the normal cells did (*t* = 6.461, *p* = 0.003), which suggests an early indication of selective cytotoxicity. This differential response became more pronounced at higher concentrations (3–300 µM). At 30 µM, a significant difference was observed (*t* = 9.128, *p* = 0.001), with MCF-7 cells showing a greater reduction in viability (72.88%) than OEC cells (86.24%), which further supports the selective anticancer activity of Zn-TCPP@GA. At 100 µM, a notable reduction in cell viability was observed in both cell types, however, the difference did not reach statistical significance (*p* = 0.082), suggesting comparable cytotoxic effects at this concentration. At the highest tested concentration (300 µM), a statistically significant difference was found (*t* = 2.534, *p* = 0.036), reinforcing the enhanced potency of the nanostructure against cancerous cells. Overall, these findings suggest that Zn-TCPP@GA exhibits a more pronounced cytotoxic effect on cancerous cells, particularly at higher concentrations, while maintaining a relatively low toxicity in normal cells, underscoring its potential as a promising and selective anticancer agent.

Numerous possible mechanisms have been proposed to explain the individual anticancer activities of Zn^2+^ ions, TCPP, and GA.^14,15,42^ TCPP, a member of the porphyrin family, has demonstrated significant anticancer potential and is the basis of clinically approved drugs recently introduced to the market such as Foscan® and Talaporfin. While TCPP is traditionally employed in PDT, this study utilized it in CDT. Here, TCPP contributes to the depletion of intracellular GSH – a key tripeptide involved in detoxification, redox balance, and cellular signalling.^43^ Moreover, TCPP serves as a structural component for MOFs, forming a porous network with possibility to embed metal ions such as Fe, Mn, Zn or Cu that can facilitate Fenton or Fenton-like reactions for ROS-based tumor destruction. Among these, zinc-based MOFs, complexes, and coordination polymers have attracted increasing interest in cancer research.^13^ Zn^2+^ ions induce anticancer effects by disrupting mitotic progression and activating caspases, the central enzymes that initiate apoptosis.^44^ Concurrently, GA demonstrates potent anticancer properties by inducing apoptosis *via* ROS generation and arresting the cell cycle at the G0/G1/M phases.^21,45^ When integrated into the Zn-TCPP@GA nanostructure, Zn^2+^ ions amplify the cytotoxic potential of both TCPP and GA, enhancing their ability to induce oxidative stress and trigger cell death in cancer cells. Collectively, the synergistic combination of three components, Zn^2+^, TCPP, and GA, contributes to the significantly enhanced anticancer efficacy of the Zn-TCPP@GA nanoplatform.

**Fig. 9:**
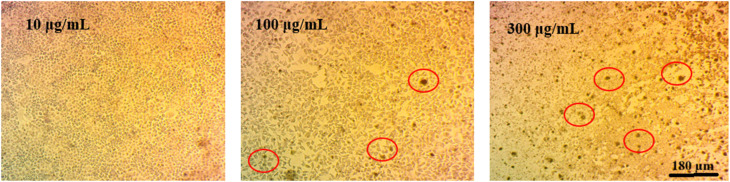
Cancer cells optical microscope images after treatment of different concentrations of Zn-TCPP@GA (10, 100, 300 µg mL^−1^).

Optical microscopy images of MCF-7 cancer cells treated with Zn-TCPP@GA at concentrations of 10, 100, and 300 µg mL^−1^ are presented in Fig. 9. A clear dose-dependent decrease in cell viability is observed, with higher concentrations of Zn-TCPP@GA leading to more pronounced morphological changes and cell death. These results underscore the enhanced cytotoxic performance of Zn-TCPP@GA, particularly at elevated doses, and highlight its better efficacy compared to cisplatin in high concentration range.

In summary, Zn-TCPP@GA demonstrated good selectivity toward cancer cells, exhibiting significantly lower cytotoxicity in normal cells, which further reinforces its favorable safety profile. The higher IC_50_ value observed in normal epithelial cells suggests reduced toxicity, positioning Zn-TCPP@GA as a promising candidate for targeted cancer therapy with minimal side effects. A key contributor to its therapeutic efficacy is the catalytic activity and ROS generating action of Zn-TCPP@GA, which plays a crucial role in its anticancer mechanism. This nanozyme-mimicking property enables Zn-TCPP@GA to catalyze the decomposition of H_2_O_2_ into ROS, inducing oxidative stress that selectively triggers apoptosis in cancer cells. Since tumor cells typically exhibit elevated endogenous H_2_O_2_ levels compared to normal tissues, Zn-TCPP@GA preferentially enhances ROS generation within malignant environments, thereby exerting selective cytotoxic effects while largely sparing healthy cells. This intrinsic catalytic function adds an important dimension to Zn-TCPP@GA's therapeutic action, offering a dual mechanism that combines direct cytotoxicity with oxidative stress-mediated apoptosis.

### Effect of synthesized Zn-TCPP@GA in MCF-7 cancer cell line apoptosis determined by flow cytometry

Flow cytometry was used to evaluate the effects of Zn-TCPP@GA on MCF-7 breast cancer cells at concentrations of 75 µg mL^−1^ and 100 µg mL^−1^, in comparison to an untreated control group (0 µg mL^−1^). In this study, MCF-7 cells were treated with Zn-TCPP@GA nanosheets for 48 hours, and the induction of apoptosis and necrosis was assessed using Annexin V-FITC and propidium iodide staining, followed by flow cytometric analysis. The percentages of necrotic cells, late apoptotic cells, early apoptotic cells, and live cells were analyzed using one-way ANOVA followed by Tukey's post hoc test, with statistical significance considered at *p* ≤ 0.05.

**Fig. 10:**
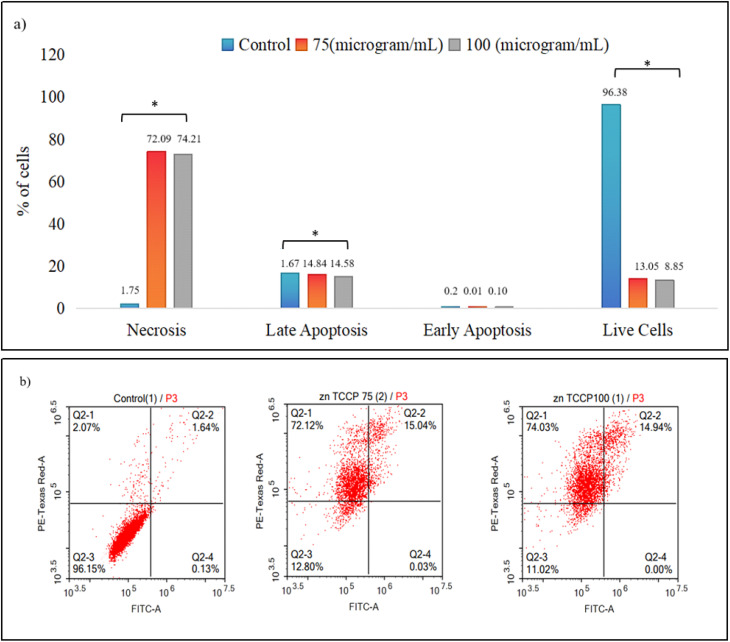
Flow cytometry for the nanostructure, (a) bars graph demonstrates the viability of cancer cells after treatment for 48 hours with 0, 75, and 100 µg mL^−1^ concentrations of Zn-TCPP@GA, (b) the apoptosis rate of tumor cells after treatment of 0, 75, 100 µg mL^−1^ for 48 h.

Fig. 10 shows a significant reduction in the viability of MCF-7 cells following treatment with 75 µg mL^−1^ of Zn-TCPP@GA, with only 13.05% of cells remaining viable. Of the treated cells, 74.09% were apoptotic, 14.58% were necrotic and 0.10% were early apoptotic, indicating a total cell death rate of around 90%. A similar pattern emerged at a higher concentration of 100 µg mL^−1^: 72.09% of cells were apoptotic, 14.84% were necrotic and 0.01% were early apoptotic. Notably, the increased necrotic cell population at 100 µg mL^−1^ suggests a possible shift towards necrotic pathways at higher concentrations or with prolonged exposure to Zn-TCPP@GA nanostructures.

The percentage of necrotic cells significantly increased with drug treatment (*F* = 14 115.92, *p* < 0.001). The untreated control group exhibited only 1.75 ± 0.29% necrosis, while treatment with 75 µg mL^−1^ and 100 µg mL^−1^ led to a marked rise in necrosis to 72.09 ± 0.96% and 74.21 ± 0.28%, respectively. Post hoc analysis showed significant differences between all groups, with completely distinct letter designations indicating statistical significance.

A similar trend was observed in late apoptosis, which also increased significantly with drug treatment (*F* = 1108.44, *p* < 0.001). The control group showed a low percentage of late apoptotic cells (1.67 ± 0.02%), while treatment with 75 µg mL^−1^ and 100 µg mL^−1^ increased this to 14.84 ± 0.58% and 14.58 ± 0.35%, respectively. Post hoc testing revealed that both treatment groups were significantly different from the control, but not significantly different from each other, as indicated by the shared letter designation. In contrast, early apoptosis did not differ significantly among the groups (*F* = 2.310, *p* = 0.180), suggesting that Zn-TCPP@GA primarily induces cell death through necrosis and late-stage apoptosis, rather than early apoptotic mechanisms.

A statistically significant reduction in the percentage of live cells was observed following drug treatment (*F* = 1436.909, *p* < 0.001). While the control group had 96.38 ± 0.20% viable cells, this percentage drastically dropped to 13.05 ± 0.46% at 75 µg mL^−1^ and further to 8.85 ± 3.87% at 100 µg mL^−1^. Post hoc analysis revealed that the two treatment groups were significantly different from the control but not from each other.

These results indicate that Zn-TCPP@GA induces cell death primarily through necrosis and late apoptosis at higher concentrations. The significant reduction in live cell populations at 75 µg mL^−1^ and 100 µg mL^−1^ suggests a dose-dependent cytotoxic effect, with minimal involvement of early apoptosis. The absence of statistical significance between the two treatment groups in late apoptosis and live cell percentages suggests that the drug reaches a saturation effect at 75 µg mL^−1^, beyond which no further increase in cytotoxicity is observed.

### Flow cytometry cell cycle

**Fig. 11:**
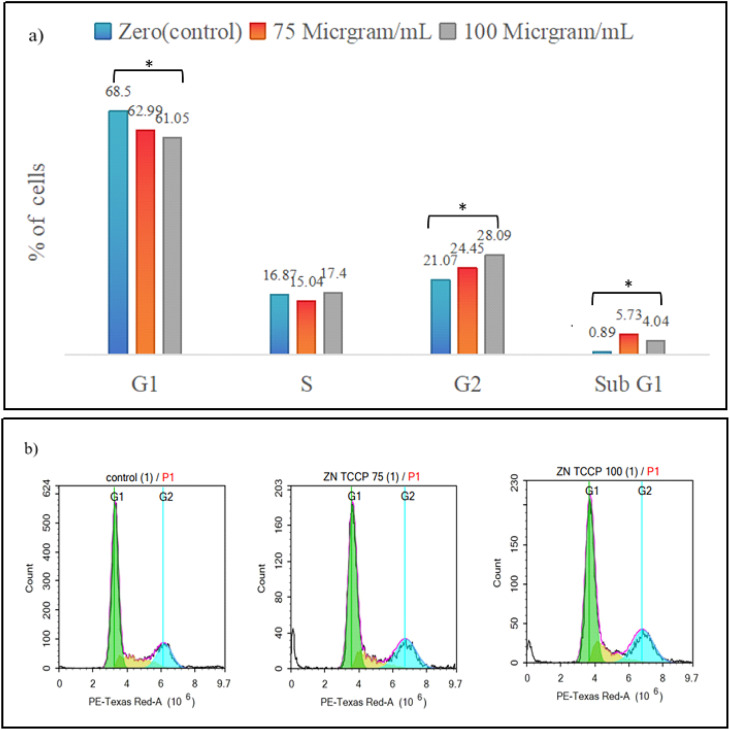
Flow cytometry for the nanostructure, (a) bars graph demonstrates the percentage of living cells during phases of cell cycle for cancer cells after treatment for 48 hours with 0, 75, and 100 µg mL^−1^ concentrations of Zn-TCPP@GA, (b) cell cycle distribution representation for tumor cells after treatment of 0, 75, 100 µg mL^−1^ for 48 h.

To evaluate the impact of Zn-TCPP@GA on cell cycle distribution and their ability to inhibit proliferation, MCF-7 cells were exposed to varying concentrations of Zn-TCPP@GA (75 and 100 µg mL^−1^) for 20 minutes in the absence of light. The treated cells were collected, fixed, and stained with propidium iodide, and flow cytometry was used to determine the distribution of cells in different cell cycle phases. When cells were treated with substances that induce apoptosis, a subpopulation of cells appeared before the G1 phase, known as the sub-G1 (apoptosis) peak. This population is believed to result from the activation of endonucleases and subsequent release of DNA from the cells. Unlike necrotic cells, apoptotic cells showed an immediate reduction in DNA content, making it possible to distinguish between the two. The extent of apoptosis in a cell population can be quantified by assessing the ratio of cells in the sub-G1 phase compared to other phases.^46^ The flow cytometry results showed a significant increase in apoptotic cells in the treated group (Table S2). Additionally, as depicted in Fig. 11, demonstrate a dose-dependent behavior in the sub-G1 population (indicating apoptotic cells).

As the dose increased, the apoptotic cells decreased. After the addition of 75 µg mL^−1^ to the cells, the sub-G1 phase increased from 0% in the control to 6.1%. However, at 100 µg mL^−1^, the sub-G1 phase decreased to 4%. In contrast, the population of non-apoptotic cells did not show significant changes. The sub-G1 cell population exhibited a substantial increase when compared to the control. Additionally, an increase in the percentage of cells in the S-phase and G2-phase was observed, indicating cell cycle arrest. Progression through the S-phase is normally controlled by replication checkpoints and DNA synthesis moderation. The increase in cells in the S-phase and G2-phase might be due to the incorporation of Zn-TCPP@GA into the damaged DNA during replication. Overall, these findings suggest that the inhibitory effect of Zn-TCPP@GA on cell growth is inducing cell death with features of apoptosis and modest G2/M accumulation, but dominated by loss of viability rather than a classical cytostatic profile. Necrosis usually occurs when the cell is exposed to certain types of cell stress, such as oxidative stress. If the cell is subjected to overwhelming levels of stress, it may die by necrosis even if it has enough energy to undergo apoptosis.^47^

The impact of the tested drug at concentrations of 75 and 100 µg mL^−1^ on cell cycle progression was evaluated using flow cytometry, and the results are summarized in Table S1. A significant reduction (*p* = 0.002) in the percentage of cells in the G1 phase was observed upon drug treatment, decreasing from 68.58% in the control to 63.0% at 75 µg mL^−1^ and 61.08% at 100 µg mL^−1^, suggesting a cell cycle arrest effect. Conversely, a significant increase (*p* = 0.031) was observed in the G2/M phase population, which rose from 21.09% in the control to 24.76% at 75 µg mL^−1^ and 28.11% at 100 µg mL^−1^, indicating a potential drug-induced G2/M arrest.

The percentage of cells in the S phase showed no statistically significant difference (*p* = 0.137) between groups, with values ranging from 16.94% in the control to 15.05% and 17.44% at 75 and 100 µg mL^−1^, respectively. Importantly, a significant increase (*p* < 0.001) was observed in the sub-G1 population, a marker of apoptotic cell death. The sub-G1 fraction increased from 0.83% in the control to 5.75% at 75 µg mL^−1^ and 4.05% at 100 µg mL^−1^, further supporting the cytotoxic effect of the drug at higher concentrations. These findings indicate that the drug induces cell cycle arrest at the G2/M phase and promotes apoptosis, as evidenced by the significant increase in the sub-G1 population.

### Intracellular oxidative stress evaluation

#### Total antioxidant capacity (TAC)

**Fig. 12:**
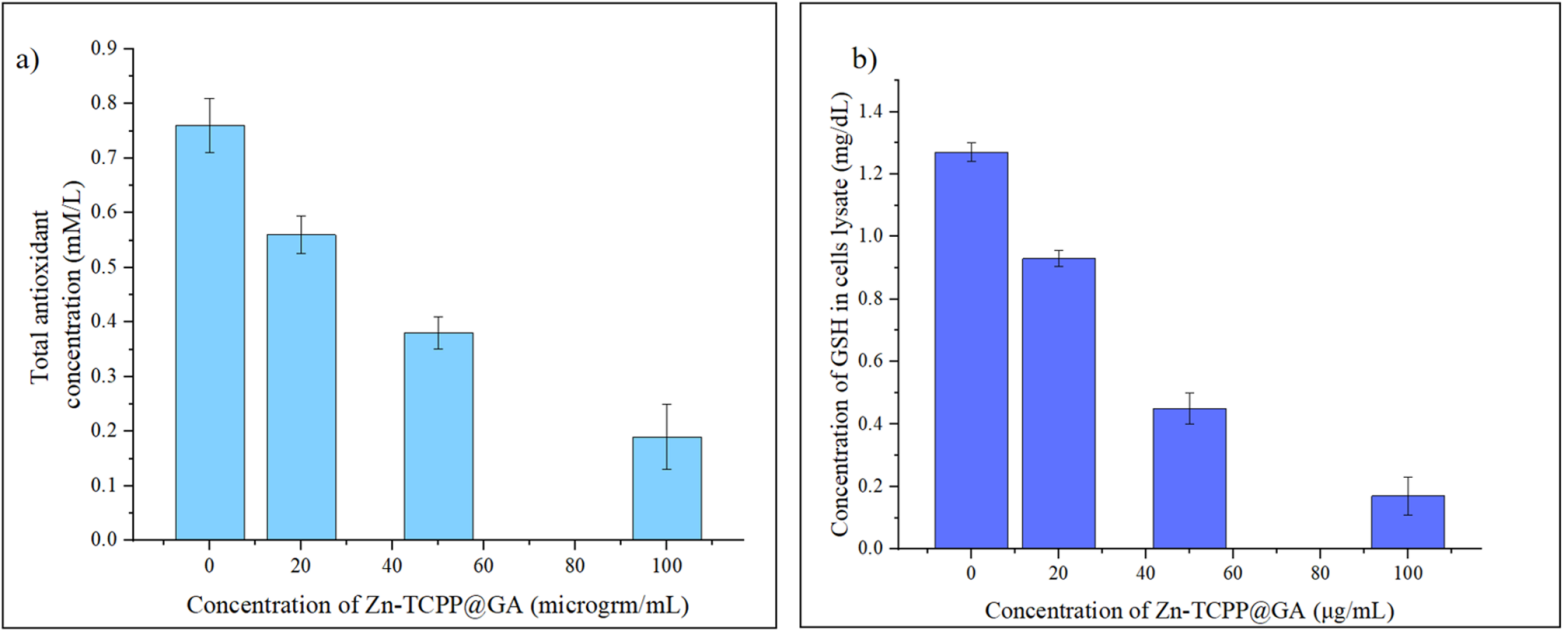
(a) TAC of cells in different Zn-TCPP@GA concentrations, (b) GSH concentration of cells treated with different Zn-TCPP@GA concentrations.

TAC provides more relevant biological information about the natural ability of cells to neutralize free radicals.^48^ It is important to emphasize that the oxidative stress done on cancer cells by the Zn-TCPP@GA will lead to decrease the ability of the cells to overcome this stress and accordingly decrease its antioxidant activity. Results are shown in Fig. 12a. Interestingly, we found a decrease in TAC in MCF-7 after 48 hours exposure to the nanosheets, which was related proportionally to the dose of the Zn-TCPP@GA. The TAC levels were significantly decreased after exposure to high conc of the nanosheets. Thus, Zn-TCPP@GA exerts promising inhibitory effects on cellular defense systems.

#### GSH depletion intracellular measurements

The intracellular GSH depletion assay is a significant tool for studying the role of GSH in cell death and other cellular processes.^49^ GSH is a tripeptide that plays a vital role in cellular protection against oxidative stress and other forms of cellular damage. GSH depletion is known to trigger cell death through a variety of mechanisms, including apoptosis, necrosis *etc.* After the addition of the Zn-TCPP@GA with different concentrations (20, 50, 100 µg mL^−1^) the consumption of GSH increased with the increase of the Zn-TCPP@GA concentration, as shown in Fig. 12b.

## Conclusions

In summary, a two-dimensional Zn-TCPP MOF was successfully synthesized *via* a straightforward one-step hydrothermal method and subsequently functionalized with GA to form the Zn-TCPP@GA nanocomposite. The resulting material demonstrated excellent potential as a chemodynamic therapy agent, primarily due to its ability to catalyze the generation of hydroxyl radicals from hydrogen peroxide under acidic, tumor-mimicking conditions, even without external light activation. This catalytic activity was significantly enhanced in the presence of H_2_O_2_ and low pH, enabling efficient ROS production, a key mechanism in CDT. In addition to ROS generation, Zn-TCPP@GA effectively depleted intracellular GSH, thereby disrupting the antioxidant defense system of cancer cells and alleviating tumor hypoxia that further amplifies the therapeutic effect. The dual functionality of ROS generation and GSH depletion positions Zn-TCPP@GA as a promising multifunctional nanoplatform for enhanced cancer treatment. These findings highlight its potential as a supplementary or combinatory therapeutic agent in next-generation oxidative stress-based cancer therapies.

Despite the promising results, further investigations are still required to provide a more comprehensive mechanistic and biological understanding of the system. Future studies should focus on obtaining more direct evidence of reactive oxygen species generation, clarifying the detailed interaction mechanism between GA and the MOF framework, and quantitatively distinguishing the contributions of different binding and catalytic pathways. In addition, more extensive surface and interface analyses would be beneficial to better elucidate the chemical nature of GA loading and its stability within the porous structure.

From a biological perspective, broader validation using additional control groups, more detailed intracellular oxidative stress measurements, and expanded apoptosis and cytotoxicity studies would further strengthen the therapeutic interpretation. Moreover, deeper insight into cellular uptake pathways, long-term biocompatibility, and *in vivo* therapeutic performance will be essential to advance this nanoplatform toward practical biomedical applications.

Overall, these future efforts will enable a more complete understanding of the structure–function relationships, catalytic behavior, and biological activity of Zn-TCPP@GA, thereby facilitating its rational optimization and translation into advanced chemodynamic cancer therapy systems.

## Conflicts of interest

There are no conflicts to declare.

## Supplementary Material

RA-016-D5RA09585A-s001

## Data Availability

The data of the entire work have been included in the bulk of the manuscript and in the supplementary information (SI) section. Supplementary information: procedures of the drug release studies, the *in silico* evaluation of the binding affinity of gallic acid to the Zn-TCPP MOF, the procedures of extracellular detection of the release of hydroxy radicals, and the cytotoxicity assays. Moreover, the data supporting this article have also been included. See DOI: https://doi.org/10.1039/d5ra09585a.
