# Supplementary material for: Porphyrin-based zinc metal–organic framework loaded with gallic acid as a novel nanoplatform exhibiting H2O2-activated reactive oxygen species generation and cytotoxicity in breast cancer cells
Source: RSC Adv. 2026 Mar 2;16(13):11727–43. doi: 10.1039/d5ra09585a (PMC12951844; doi:10.1039/d5ra09585a)
Supplement: RA-016-D5RA09585A-s001 [file RA-016-D5RA09585A-s001.pdf]

Supplementary Information (SI) for RSC Advances.
This journal is © The Royal Society of Chemistry 2026

# Supplementary Information

## Drug Release Studies

An amount of 10 mg of dry Zn-TCPP@GA was placed in a dialysis bag then soaked and stirred in 20 mL of PBS at room temperature for drug release studies. A 5 mL aliquot of mixed solution was collected every hour for the first 6 hours, followed by one sample collected on daily basis for the following seven days. The collected aliquots were filtered through a syringe filter (PVDF, 0.45 µm, 13 mm diameter) to remove remaining Zn-TCPP remains before analysis. GA release amounts and Zn-TCPP degradation rates were examined by monitoring the changes in concentrations of GA and TCPP over time using UV-Vis spectroscopy. Additionally, to test the release of zinc ions ICP-MS analysis was used to detect The $Zn^{2+}$ ion concentrations in each time intervals. All measurements were carried out in triplicates and results were plotted as mean values ± standard deviation. The release kinetics of the system were evaluated using different drug delivery models, including zero-order, first-order, Hixson−Crowell, and Higuchi models., where a higher correlation coefficient confirms that the drug delivery system follows the corresponding kinetic model. A zero-order drug delivery model is commonly used to explain the diffusion behaviors of a system and can be expressed in equation 1. [25].

$$\frac{M_t}{M_\infty} = kt \qquad (1)$$

Where, $M_t$ and $M_\infty$ represent the absolute cumulative amount of released drug at a specific time t and at infinite time, respectively, and $k$ is the release rate constant. The ratio of $M_t$ and $M_\infty$ represents the cumulative drug release percent. The first-order kinetic equation takes consideration of the material degradation in water and can be expressed in equation (2).

$$\ln\left(1 - \frac{M_t}{M_\infty}\right) =- kt \qquad (2)$$

The Hixson−Crowell model was also applied to consider a bulk-erosion system since Zn-TCPP may degrade in physiological environment. The Hixson−Crowell model can be expressed in equation (3).

$$\sqrt[3]{\left(1-\frac{M_t}{M_\infty}\right)}=-kt \quad (3)$$

The Peppas model is generally used for modeling polymer-based drug delivery systems with surface erosion. It can be expressed in equation (4).

$$\frac{M_t}{M_\infty}=kt^n \quad (4)$$

Where n is the release exponent.

The Higuchi model was also applied to fit the cumulative drug release percent as a function of time. This model takes into consideration the controlled release of the drug by diffusion and degradation processes of porous matrices and can be expressed in equation (5) [26].

$$\frac{M_t}{M_\infty}=k_h\sqrt{t} \quad (5)$$

where $M_t / M_\infty$ is the fraction of drug released at each time point (t), $M_t$ is the amount of drug released in time t, $M_\infty$ is the amount of drug released after time ∞, and $k_h$ represents the Higuchi release kinetic constant. Here, the plot is obtained by cumulative percentage drug release vs. square root of time (t). As the drug diffuses out of the matrix, the concentration gradient between the inside and outside of the matrix decreases. This decrease in concentration gradient leads to a decrease in the rate of drug release. However, the rate of drug release is still proportional to the square root of time (t) because the diffusion process is a function of the concentration gradient.

**In Silico Evaluation of Gallic Acid Binding Affinity toward Zn-TCPP MOF**

A 100-ns molecular dynamics (MD) simulation was performed to investigate the behavior of gallic acid within the Zn-TCPP MOF pore immersed in explicit water containing 0.15 M NaCl. All simulations were executed using the Desmond software package (Schrödinger Inc.) [27] in the isothermal-isobaric (NPT) ensemble at 300 K and 1 atm. The MOF framework was treated as rigid, with all framework atoms fixed in space to reduce computational expense-an acceptable approximation for robust MOF with minimal framework flexibility. The gallic acid molecule

(3,4,5-trihydroxybenzoic acid) was modeled in its deprotonated form (carboxylate anion), appropriate for neutral pH conditions, and parameterized using the OPLS-AA force field. [28] Water molecules were represented using the three-site SPC model, and ions were added to mimic physiological ionic strength. Long-range electrostatics were computed via the particle-mesh Ewald (PME) method with a 9 Å real-space cutoff. [29]Temperature and pressure were maintained with a Nosé-Hoover chain thermostat and a Martyna-Tuckerman-Klein barostat, respectively. A 2 fs time step was employed, with all bonds involving hydrogen atoms constrained.

Geometric analysis of the Zn-TCPP MOF's pore structure was performed using Zeo++ [30], a pore characterization software. The crystal structure (periodic cell) of the dry Zn-TCPP framework was analyzed to determine accessible surface area, pore volume, and pore size distribution. The accessible internal surface area was estimated on the order of a few hundred $m^2/g$, consistent with literature reports for similar TCPP-based MOFs (typically 330–600 $m^2/g$) [31]. These analyses confirmed that the pores are large enough to accommodate a gallic acid molecule and that the framework maintains permanent porosity in the absence of guests.

To evaluate the uptake capacity of gallic acid in the Zn-TCPP framework under equilibrium conditions, grand canonical Monte Carlo (GCMC) simulations [32] were conducted using the RASPA 2.0 software [33] package. Simulations were performed in the μVT ensemble, maintaining constant chemical potential (μ), volume (V), and temperature (T), and were designed to emulate the adsorption of gallic acid from a saturated aqueous solution. The MOF was modeled as rigid during these simulations, and interactions were described using Lennard-Jones (12–6) plus Coulombic potentials. Gallic acid was assigned OPLS-AA parameters, while the MOF was modeled using UFF parameters. Ewald summation was used for electrostatics, and van der Waals interactions were truncated at 12.0 Å.

Each GCMC simulation consisted of 10,000 equilibration cycles followed by 10,000 production cycles. The sampling moves included trial insertions, deletions, rotations, and translations of gallic acid molecules. The resulting uptake was expressed in gravimetric terms (mg of gallic acid per gram of MOF), calculated using equation (6):

$$U = \frac{N_{\text{ads}} \times M_{\text{GA}}}{N_A \times m_{\text{MOF}}} \quad (6)$$

Where:

- $N_{ads}$ is the number of adsorbed gallic acid molecules per simulation box,
- $M_{GA}$ is the molar mass of gallic acid (170.12 g/mol),
- $N_A$ is Avogadro's number,
- $m_{MOF}$ is the total mass of the MOF within the simulation box.

This approach yields the theoretical maximum loading under idealized conditions, where guest–framework interactions dominate in the absence of solvent competition.

To assess the diffusional mobility of gallic acid within the MOF pores, the mean square displacement (MSD) was calculated from the MD trajectory. This metric quantifies the average squared displacement of the gallic acid molecule's center of mass over time and is defined by equation (7):

$$\mathrm{MSD}(t) = \left\langle \left|\vec{r}_i(t) - \vec{r}_i(0)\right|^2 \right\rangle \tag{7}$$

where $r_i$ ($t$) is the position vector of the molecule at time $t$, and the angle brackets indicate a time-averaged value over multiple origins. A linear increase in MSD with time is indicative of normal diffusion, whereas a sub-linear or plateauing trend reflects constrained or immobilized behavior due to strong host–guest interactions. Trajectory data were sampled every 1 ps, and analyses were carried out using in-house Python scripts integrated with the MDAnalysis and Matplotlib libraries.

To further elucidate the local structural environment and interaction specificity, the radial distribution function (RDF), $g_{ij}(r)$, was computed between the carboxylate oxygen atoms of gallic acid and the $Zn^{2+}$ nodes in the MOF framework. RDF provides a measure of the probability of finding an atom j at a distance r from atom i, relative to an ideal gas distribution, and is defined by equation (8):

$$g_{ij}(r) = \frac{1}{4\pi r^2 \rho_j} \left\langle \frac{dn_{ij}(r)}{dr} \right\rangle \tag{8}$$

Here, $dn_{ij}$ (r) is the number of atom $j$ located within a shell $dr$ at distance $r$ from atom $i$, and $\rho_j$ is the average bulk number density of atom type $j$. A sharp and intense first peak at short distance (typically 2.5–2.7 Å) is indicative of direct coordination or strong electrostatic interaction. A broader secondary peak may indicate secondary coordination shells or extended structural

correlations. RDF profiles were generated from 10,000 frames extracted at 1 ps intervals from the equilibrated portion of the MD trajectory.

## Extracellular Detection of ·OH Radical Release

### MB Colorimetric Method

This assay was performed to detect extracellular •OH. Zn-TCPP@GA (0.6 mg) were added to 3 mL of MB solution (6 mM) to establish an adsorption/desorption equilibrium in the dark for 1h. Then, Zn-TCPP@GA nanosheets with different concentrations (0.6 mL, 0.1, 0.2, 0.3 and 0.4 mg/mL) were dispersed into a MB solution (6 mM)w222. A standard solution of $H_2O_2$ with the concentration of $1\times10^{-3}$ mol/L was prepared by dilution of a 30% $H_2O_2$ solution (Sigma-Aldrich). Then, 1 mL of $H_2O_2$ solution was added into the Zn-TCPP@GA and MB solutions. The absorbance at 663 nm was measured by UV−vis spectroscopy (34).

### Terephthalic Acid Fluorescence Method

Hydroxyl radicals were detected by using disodium terephthalate as a capture agent, which can react with hydroxyl radicals to generate fluorescent 2-hydroxyterephthalic disodium as mentioned before [35] with slight modifications. Briefly, terephthalic acid (TA, $6\times10^{-2}$ M) was dissolved in NaOH ($2\times10^{-2}$ M) solution. In a typical process, Zn-TCPP@GA nanosheets (0.6 mL, 0.1, 0.2, 0.3 and 0.4 mg/mL) were dispersed in a TA solution (6 mM). A standard solution of $H_2O_2$ with the concentration of $1 \times 10^{-3}$ mol/L was prepared from a 30% solution (Sigma-Aldrich) by dilution. Then, 1 mL of $H_2O_2$ solution was added to the (TA+Zn-TCPP@GA) solution and shaked for one hour. Finally, the fluorescence spectra of TA were collected at 426 nm and were plotted as a function of the Zn-TCPP@GA concentration.

## Cytotoxicity Assay

### Cell Culture

Breast Adenocarcinoma cells (MCF-7) were obtained from Nawah Scientific Inc., (Mokatam, Cairo, Egypt). Cells were maintained in DMEM media supplemented with 100 mg/mL of streptomycin, 100 units/mL of penicillin and 10% of heat-inactivated fetal bovine serum in humidified, 5% (v/v) $CO_2$ atmosphere at 37 °C.

**Cell Viability**

Cell viability was assessed by 3-(4,5-dimethylethiazole-2-yl)-2, 5-diphynyltetrazolium bromide assay (MTT). Aliquots of 100 µL cell suspension ($5x10^3$ cells) were seeded in 96-well plates and incubated in complete media for 24 h at 37°C in a 5% $CO_2$ incubator. Cells were treated with another aliquot of 100 µL media containing Zn-TCPP@GA at various concentrations. After 48 h of drug exposure, media was discarded then MTT solution (20 µL of 1 mg/mL stock solution was added to 100 µL of PBS in each well and incubated at 37°C for 4 h. The formed formazan crystals were then dissolved in 100 µL of absolute DMSO. The absorbance of formazan solutions was measured at $\lambda_{max}$ 570 nm using a multi-well plate reader (BMGLABTECH®FLUOstar Omega, Germany). Statistical analysis was conducted using GraphPad Prism 6.0 software. Differences between groups were evaluated using Student's t-test. Each assay was performed in triplicate and conducted in parallel, with results expressed as mean ± standard deviation. A *p*-value of less than 0.05 was considered statistically significant.

**Flowcytometry Assay**

Apoptosis and necrosis of cell populations were determined using Annexin V-FITC apoptosis detection kit (Abcam Inc., Cambridge Science Park, Cambridge, UK) coupled with 2 fluorescent channels flowcytometry. After treatment with test compounds for the specified duration, cells ($10^5$ cells) were collected by trypsinization and washed twice with ice-cooled PBS (pH 7.4). Then, cells were incubated in the dark with 0.5 ml of Annexin V-FITC/PI solution for 30 min at room temperature according to manufacturer's protocol. After staining, cells were injected via ACEA Novocyte™ flowcytometer (ACEA Biosciences Inc., San Diego, CA, USA) and analyzed for FITC and PI fluorescent signals using FL1 and FL2 signal detectors, respectively ($\lambda_{ex/em}$ 488/530 nm for FITC and $\lambda_{ex/em}$ 535/617 nm for PI). For each sample, 12,000 events were acquired and positive FITC and/or PI cells were quantified by quadrant analysis and calculated using ACEA NovoExpress™ software (ACEA Biosciences Inc., San Diego, CA, USA).

After treatment with test compounds for the specified duration, cells ($10^5$ cells) were collected by trypsinization and washed twice with ice-cooled PBS (pH 7.4). Cells were resuspended in 2 mL of 60% ice-cooled ethanol and incubated at 4°C for 1 h for fixation. Fixed cells were again washed twice with PBS (pH 7.4) and re-suspended in 1 mL of PBS containing 50 µg/mL RNAase A and 10 µg/mL propidium iodide (PI). After 20 min of incubation in dark at 37

°C, cells were analyzed for DNA contents using flow cytometry analysis using FL2 ($\lambda_{ex/em}$ 535/617 nm) signal detector (ACEA Novocyte™ flow cytometer, ACEA Biosciences Inc., San Diego, CA, USA). For each sample, 12,000 events were acquired. Cell cycle distribution was calculated using ACEA NovoExpress™ software (ACEA Biosciences Inc., San Diego, CA, USA).

**Intracellular Oxidative Stress Evaluation and Total Antioxidant Capacity (TAC)**

TAC test was performed on cancer cells after treatment with Zn-TCPP@GA to assess the cancer cell's ability to defend itself against oxidative stress. To ensure that cancer cells often have higher levels of oxidative stress than normal cells, a TAC test was carried out. Briefly, the TAC was determined using a commercially available kit (Biodiagnostic, Egypt). according to the manufacturer's instructions, with modifications. The cells were cultured in a 10 cm petri dish until they reached 80%–90% confluence and treated as indicated in the manufacturer protocol. For determining cellular TAC, post-Zn-TCPP@GA treated cells were washed with PBS and suspended in 200 μL of ice-cooled lysis buffer and sonicated. The lysate was centrifuged at 10,000 RPM for 10 minutes. A certain amount of the substrate, exogenously provided hydrogen peroxide, was eliminated with the total antioxidants within the sample and the chromogen (3,5, dichloro-2-hydroxy benzenesulfonate) was converted to a colored product by enzymatic reaction included the residual of $H_2O_2$. This colored product was measured at 505 nm using a plate reader (BMG Labtech, FLUOstar Omega, Germany). Absorbance values were measured for 3 different Zn-TCPP@GA concentrations.

**GSH Depletion Test**

To measure the ability of the Zn-TCPP@GA 2D MOF to deplete GSH inside cells, the intracellular GSH depletion test was performed on three different Zn-TCPP@GA concentrations according to the method mentioned before [24] with some modifications. Briefly, according to the manufacturer's instructions, the GSH contents in these treated cells were tested by using the GSH assay commercial kit that was used for the analysis (Bio Diagnostic, Egypt). MCF-7 cells were seeded in culture bottles for 24 h (37°C, 5% $CO_2$). Then, MOF solutions (containing different concentrations in medium) were added and incubated for 24 h. After that, each culture bottle was rinsed with PBS and centrifuged at 3000 RPM to collect the MCF-7 cells. After that, 0.5 mL of the cells were added to 2 mL of the kit-DNTB (5,5`dithiobis (2-nitrobenzoic acid), which was reduced with glutathione (GSH) within the sample to produce the yellow TNB (5-thio-2-

nitrobenzoic acid). The reduced chromogen's absorbance was measured at 405 nm using a plate reader (BMG Labtech, FLUOstar Omega, Germany). and was directly proportional to the GSH content within the sample. One-way ANOVA with Tukey's post hoc test was used. $p \leq 0.05$ was considered significant.

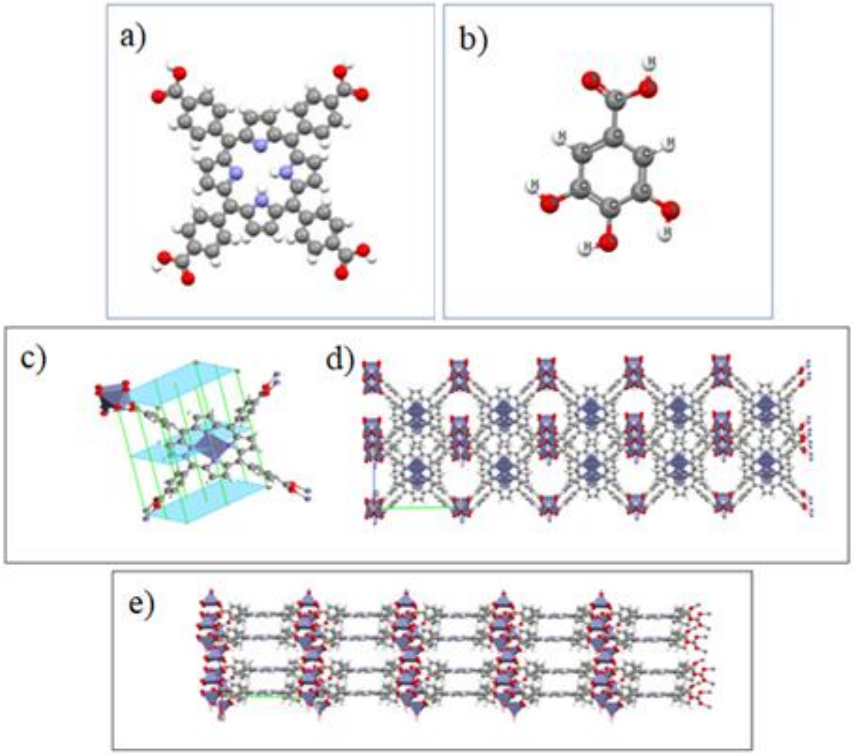


**Figure S1**. Chemical structure of GA (a), Chemical structure of TCPP (b), A Secondary building unite (SBU) of Zn-TCPP MOF showing the binding between the Zn(II) ions and the TCPP linker molecules (c), The crystal structure of Zn-TCPP MOF. Color codes: C, grey; N, sky blue; O, red; Zn, blue; H, white.

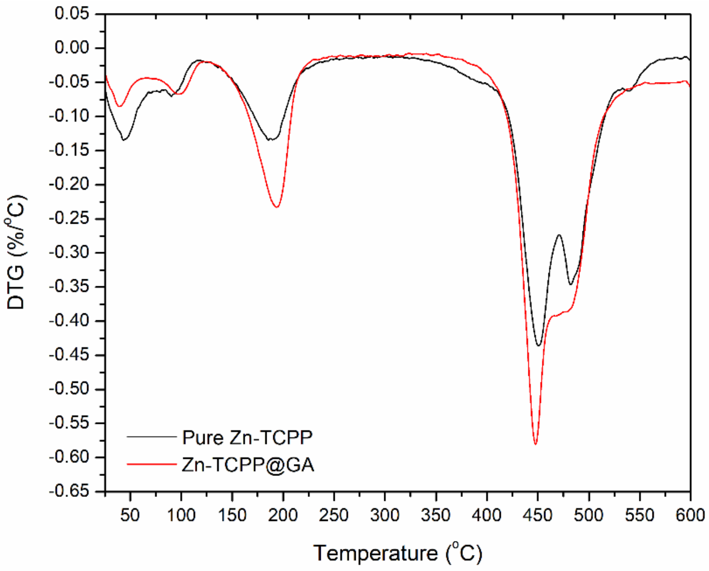


**Figure S2**. DTG thermograms of Zn-TCPP pure MOF and Zn-TCPP@GA structures.

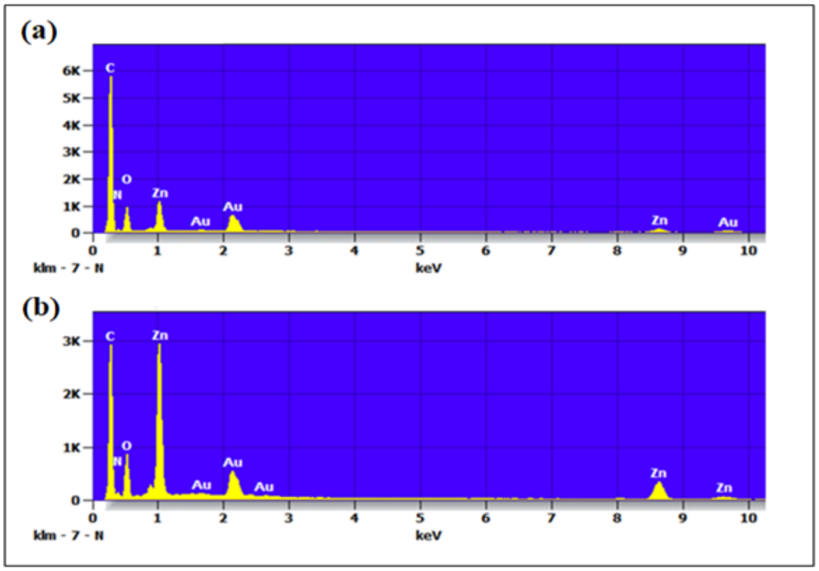


**Figure S3**. EDX spectra of Zn-TCPP (a) and Zn-TCPP@GA (b).

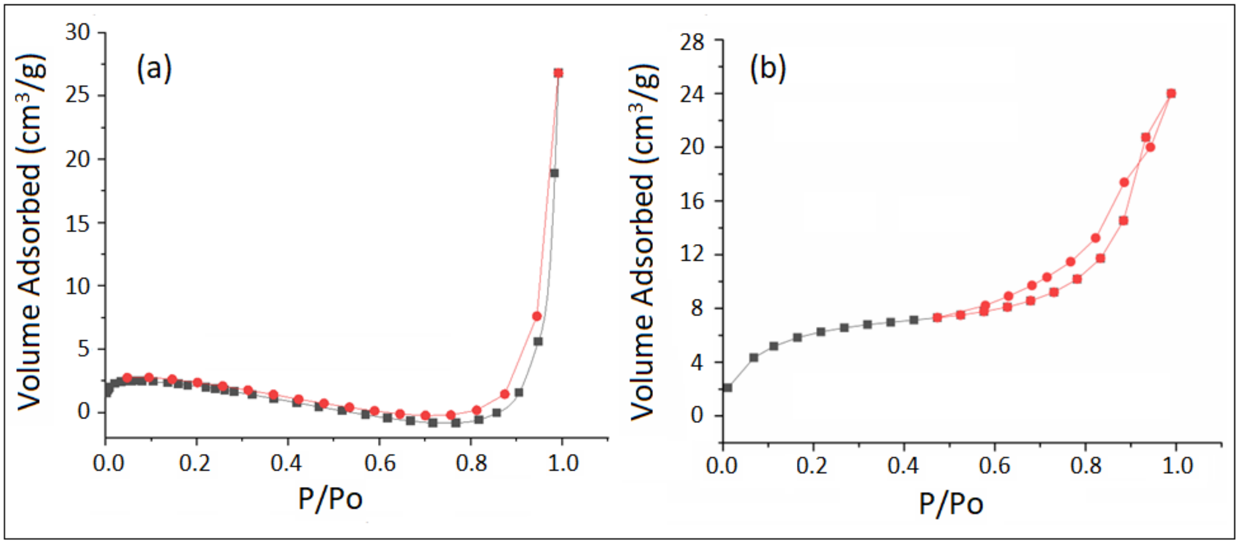


**Figure S4**. $N_2$-adsorption hysteresis of a) as-prepared Zn-TCPP MOF structure b) after GA loading

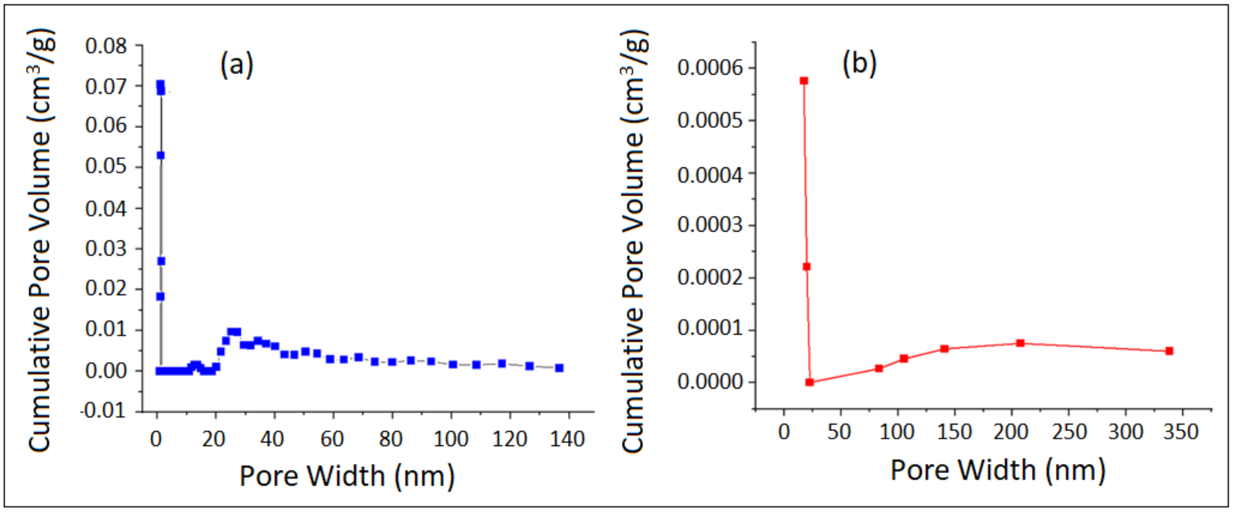


**Figure S5**. Pore-size distribution of the a) as-prepared Zn-TCPP MOF structure, b) after GA loading

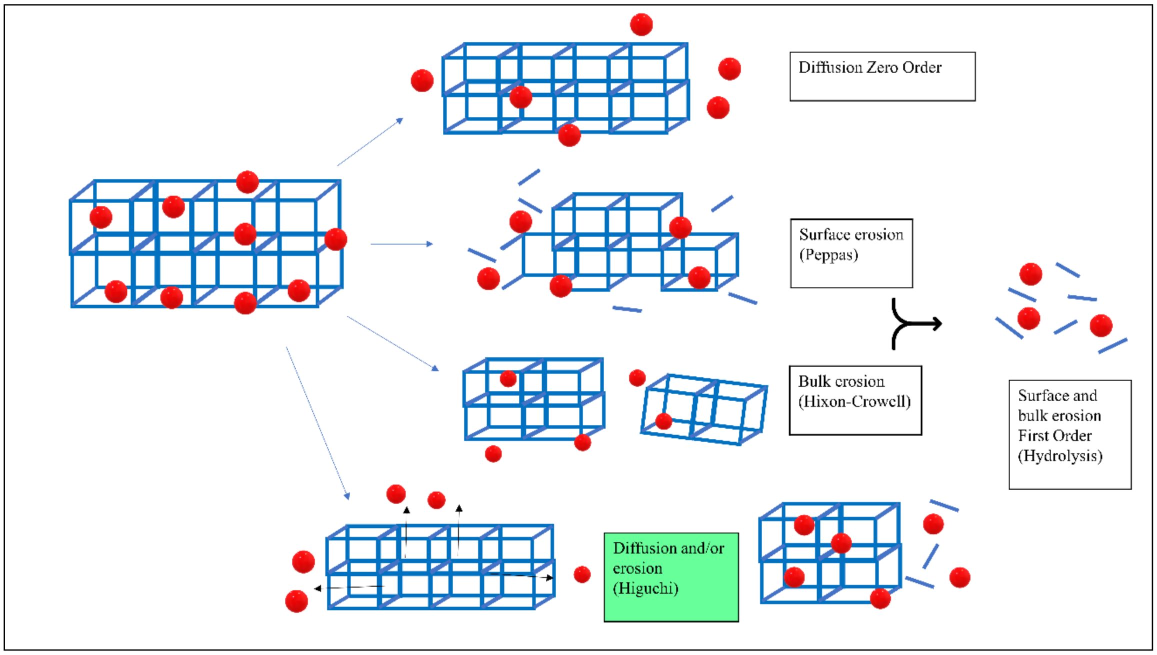


**Figure S6**. Schematic demonstrating the different kinetics of therapeutic agent release.

**Table S1**. Comparison between the three Doses on % Cell Population

| | Zero (control) | 75 Micrgram/mL | 100 Micrgram/mL | F | p |
|---|---|---|---|---|---|
| **Necrosis** | $1.75^{c} \pm 0.29$ | $72.09^{b} \pm 0.96$ | $74.21^{a} \pm 0.28$ | $14115.92^{*}$ | $<0.001^{*}$ |
| **Late apoptosis** | $1.67^{b} \pm 0.02$ | $14.84^{a} \pm 0.58$ | $14.58^{a} \pm 0.35$ | $1108.436^{*}$ | $<0.001^{*}$ |
| **Early apoptosis** | $0.20^{a} \pm 0.07$ | $0.01^{a} \pm 0.02$ | $0.10^{a} \pm 0.17$ | 2.310 | 0.180 |
| **Live cells** | $96.38^{a} \pm 0.20$ | $13.05^{b} \pm 0.46$ | $8.85^{b} \pm 3.87$ | $1436.909^{*}$ | $<0.001^{*}$ |

**Table S2**. Comparison between the three Doses according to % Cell Cycle Phases

| | Zero (control) | 75 Micrgram/mL | 100 Micrgram/mL | F | p |
|---|---|---|---|---|---|
| **G1** | $68.58^{a} \pm 1.03$ | $63.0^{b} \pm 1.05$ | $61.08^{b} \pm 2.22$ | $19.257^{*}$ | $0.002^{*}$ |
| **S** | $16.94^{a} \pm 1.71$ | $15.05^{a} \pm 0.47$ | $17.44^{a} \pm 1.38$ | 2.815 | 0.137 |
| **G2** | $21.09^{b} \pm 0.93$ | $24.76^{ab} \pm 3.81$ | $28.11^{a} \pm 1.23$ | $6.557^{*}$ | $0.031^{*}$ |
| **Sub G1** | $0.83^{c} \pm 0.10$ | $5.75^{a} \pm 0.43$ | $4.05^{b} \pm 0.30$ | $200.018^{*}$ | $<0.001^{*}$ |
